# Wild birds drive the introduction, maintenance, and spread of H5N1 clade 2.3.4.4b high pathogenicity avian influenza viruses in Spain, 2021–2022

**DOI:** 10.1093/ve/veag006

**Published:** 2026-01-30

**Authors:** Andrew Y Cho, Dong-Hun Lee, Alice Fusaro, Edoardo Giussani, Ambra Pastori, Montserrat Agüero, Natàlia Majó, Kateri Bertran

**Affiliations:** Avian Disease Laboratory, College of Veterinary Medicine, Konkuk University, 120 Neungdong-ro, Gwangjin-gu, Seoul 05029, Republic of Korea; Wildlife Health laboratory, College of Veterinary Medicine, Konkuk University, 120 Neungdong-ro, Gwangjin-gu, Seoul 05029, Republic of Korea; Konkuk University Zoonotic Diseases Research Center, College of Veterinary Medicine, Konkuk University, 120 Neungdong-ro, Gwangjin-gu, Seoul 05029, Republic of Korea; European Union Reference Laboratory (EURL) for Avian Influenza and Newcastle Disease, Istituto Zooprofilattico Sperimentale delle Venezie, viale dell'universita 10, Legnaro, Padua 35020, Italy; European Union Reference Laboratory (EURL) for Avian Influenza and Newcastle Disease, Istituto Zooprofilattico Sperimentale delle Venezie, viale dell'universita 10, Legnaro, Padua 35020, Italy; European Union Reference Laboratory (EURL) for Avian Influenza and Newcastle Disease, Istituto Zooprofilattico Sperimentale delle Venezie, viale dell'universita 10, Legnaro, Padua 35020, Italy; Laboratorio Central de Veterinaria, Ministry of Agriculture, Fisheries and Food, Ctra. M-106, Km 1,4 Algete 28110, Madrid, Spain; Unitat mixta d’Investigació IRTA-UAB en Sanitat Animal, Centre de Recerca en Sanitat Animal (CReSA), Edifici CReSA, Campus de la Universitat Autònoma de Barcelona (UAB), Bellaterra 08193, Catalonia, Spain; Departament de Sanitat i Anatomia Animals, Facultat de Veterinària, Travessera dels Turons s/n, Campus de la Universitat Autònoma de Barcelona (UAB), Bellaterra 08193, Catalonia, Spain; Unitat mixta d’Investigació IRTA-UAB en Sanitat Animal, Centre de Recerca en Sanitat Animal (CReSA), Edifici CReSA, Campus de la Universitat Autònoma de Barcelona (UAB), Bellaterra 08193, Catalonia, Spain; IRTA, Programa de Sanitat Animal, Centre de Recerca en Sanitat Animal (CReSA), Edifici CReSA, Campus de la Universitat Autònoma de Barcelona (UAB), Bellaterra 08193, Catalonia, Spain

**Keywords:** H5N1, wild birds, Bayesian, phylogenetics, Spain, highly pathogenic avian influenza

## Abstract

The 2021–2022 high pathogenicity avian influenza (HPAI) epizootic was the worst ever recorded in Europe in general and in Spain in particular. Between December 2021 and November 2022, H5N1 clade 2.3.4.4b HPAI viruses caused outbreaks in both wild birds and domestic poultry in Spain. We analysed the complete genome sequences of H5N1 HPAI viruses identified during this period in Spain and conducted comparative phylogenetic analyses to identify their origin and reconstruct their evolutionary and diffusion dynamics. We identified four different genetic reassortants of H5N1 clade 2.3.4.4b HPAI viruses. Our results suggest multiple wild bird introductions of H5N1 clade 2.3.4.4b HPAI viruses into different regions of Spain from other European countries. Bayesian phylodynamic analyses of H5N1 clade 2.3.4.4b HPAI viruses support that their initial entry into Spain occurred in the North-West and South-West through wild birds, which further spread the viruses to other regions within Spain. Andalusia (South) was the hotspot for maintenance of viruses in poultry. Wild Anseriformes played a crucial role in the introduction of the viruses into Spain and the subsequent transmission of these viruses to other host types of birds, both wild and domestic. This study highlights the role of wild birds in the ecology of H5N1 clade 2.3.4.4b HPAI viruses and provides further insight into the genetic diversity, evolution, and spread of these viruses between wild birds and poultry.

## Introduction

Since its emergence in 1996 in Guangdong, China, high pathogenicity avian influenza (HPAI) viruses of A/goose/Guangdong/1/1996 (Gs/GD) lineage have spread globally infecting domestic and wild birds and occasionally spilling over into mammals, including humans (Xu et al. 1999, Chen et al. 2005, Lee et al. 2017, Antigua et al. 2019). Over time, the H5Nx HPAI Gs/GD lineage has diverged into multiple phylogenetically distinct clades, bearing variable gene segment constellations by reassortment with low pathogenic avian influenza (LPAI) viruses (Gu et al. 2011, Wu et al. 2014, Bi et al. 2016, Lee et al. 2017, WHO 2021). The H5 clade 2.3.4.4 has become dominant and further diversified genetically into subclades a–h (WHO 2021). The related H5 clade 2.3.4.4b HPAI viruses have circulated in the wild bird population and, unlike previous HPAI viruses, have been maintained in these species for a long time, changing the epidemiology of HPAI viruses (Lee et al. 2017, Bodewes and Kuiken 2018, Pohlmann et al. 2022). Multiple genotypes with gene segments of wild bird-origin LPAI viruses have been detected mainly in Eurasia (Poen et al. 2019, Jeong et al. 2020, King et al. 2020, Fusaro et al. 2024). Between 2016 and 2022, repeated waves of reassortant H5Nx clade 2.3.4.4b HPAI viruses in Europe have caused large numbers of mortality events and outbreaks among free-living wild birds, poultry, and other captive birds in Europe (Alarcon et al. 2018, Napp et al. 2018, Verhagen et al. 2021, EFSA et al. 2023b).

The 2021–2022 HPAI epizootic was the largest ever recorded in the European continent, with an unprecedented number of affected wild and domestic bird populations, high mortality rates, and an unparalleled increased susceptibility of mammalian species (EFSA et al. 2022a). In addition, the virus circulation in wild birds throughout the year affecting not only migratory but also resident birds during summer months, and the unusual geographical extent of H5N1 clade 2.3.4.4.b HPAI viruses posed a new threat for new vulnerable host species and countries (EFSA et al. 2022a). One of the unique features of this epizootic was HPAI virus circulation in Southern European countries, traditionally much less affected than Central and Northern countries (EFSA et al. 2022a). While Spain was not affected in the previous epizootic seasons, a total of 138 outbreaks (defined as confirmed detections in one or more individuals per event) in wild birds (135 outbreaks in free-living birds and 3 outbreaks in captive birds) and 36 outbreaks (defined at the farm or premises level) in poultry were reported between December 2021 and November 2022, representing the worst HPAI season ever recorded in Spain (EFSA et al. 2022a).

There are still significant knowledge gaps regarding the connections between wild birds and poultry holdings, as well as the role of wild birds and poultry production in the dissemination and progression of H5N1 HPAI viruses in Spain. In this study, we analysed the complete genome sequences of H5N1 HPAI viruses identified during the 2021–2022 season in Spain. Comparative phylogenetic analyses were conducted to identify the origin and reconstruct the evolutionary and diffusion dynamics of the H5N1 HPAI viruses in Spain.

## Materials and methods

### Sampling for AIV detection and whole genome sequencing

A total of 36 outbreaks in poultry, 135 outbreaks in free-living wild birds, and 3 outbreaks in captive birds were reported between December 2021 and November 2022 in Spain (https://servicio.mapa.gob.es/rasve/Publico/Publico/BuscadorFocos.aspx). Viral RNA extracted from tracheal or cloacal swabs tested positive for H5Nx HPAI virus by standard RT-qPCR methods in the National Reference Laboratory for AIV (Laboratorio Central Veterinario-Sanidad Animal, Algete, Madrid, Spain) (Spackman et al. 2003, Agüero et al. 2007, Slomka et al. 2007, Heine et al. 2015, Hoffmann et al. 2016). Viral RNA samples were sequenced on an Illumina® MiSeq platform (San Diego, CA) at Istituto Zooprofilattico Sperimentale delle Venezie (IZSVe), Padua, Italy, as previously described (Fusaro et al. 2019).

Trimmomatic v0.32, scythe v0.991 (https://github.com/vsbuffalo/scythe) and sickle v1.33 (https://github.com/najoshi/sickle) were used to trim low quality 3′ bases (<Q20) and Nextera XT adapters. Reads shorter than 80 bases or unpaired after previous filters were discarded (Bolger et al. 2014). BWA v0.7.1229 was used to align the cleaned reads against a reference genome (Li 2013). Picard tools v2.1.0 (https://broadinstitute.github.io/picard/) and GATK v3.530–32 were applied to correct potential errors, realign reads around indels and recalibrate base quality in alignments. LoFreq v2.1.233 was used for variant calling and the consensus sequences were produced with an *in-house* script (Wilm et al. 2012, O'Connor and van der Auwera 2020). The complete genome sequences of a total of 124 viruses out of the total 174 confirmed outbreaks from Spain analysed in this study are available in GISAID under accession numbers found in the Supplementary Table S1.

### Genotyping

To examine the phylogenetic relationships of the sequenced isolates, homologous sequences were identified and downloaded from the GISAID database using BLAST query. Maximum likelihood (ML) trees of each gene segment, aligned along with the downloaded sequences using the MAFFT v7.453 software, were constructed using the RAxML v8.0 through 1 000 bootstrap using general time-reversible (GTR) + Gamma model (Yang 1994, Katoh and Standley 2013, Stamatakis 2014). Resulting phylogenetic trees were visualized using the online tool iTOL v5 (https://itol.embl.de/) (Letunic and Bork 2021). Monophyletic clades with bootstrap support of 70% and higher were considered well supported (Hillis and Bull 1993). Genotypes were defined when the ML phylogenies of the eight segments of avian influenza (AI) virus segments showed a unique genome constellation for one or more isolates, as previously described (Fusaro et al. 2019).

### Nextstrain build of H5N1 clade 2.3.4.4b HPAI in Spain

To understand the H5N1 clade 2.3.4.4b HPAI 2021–2022 epizootic in Europe, Nextstrain build was designed with the hemagglutinin (HA) sequences downloaded from GISAID database by the following criteria: “Type A,” “H5,” “Host Avian,” “Location Europe,” and “Collection date from October 1^st^, 2021 to December 31^st^, 2022” (Shu and McCauley 2017, Hadfield et al. 2018). Nextstrain build was generated using the Augur pipeline (https://github.com/nextstrain/augur), which incorporates MAFFT for sequence alignment and TreeTime for molecular clock inference and ancestral trait reconstruction (Katoh and Standley 2013, Sagulenko et al. 2018). The build was executed with default settings and visualized using Auspice (https://docs.nextstrain.org/projects/auspice). The Nextstrain build for Spain and the rest of Europe during 2021–2022 is uploaded on Nextstrain public group (https://nextstrain.org/groups/KU-song-public). Frequencies of the viral isolates were calculated at 2-week intervals using a narrow bandwidth of 0.041, with no wide-bandwidth smoothing applied**.**

### Ancestral state reconstruction of geographic location and host type

Time-measured phylogeny of the HA gene was inferred using the software BEAST v1.10.4 (https://beast.community) and BEAGLE library (Suchard et al. 2018, Ayres et al. 2019). A subset of HA sequences including the isolates from Spain was selected from the initial ML phylogeny for further analysis. Root-to-tip regression analysis was conducted using TempEst v1.5.3 (http://tree.bio.ed.ac.uk/software/tempest/) to assess the temporal signal (R^2^ > 0.5) of the dataset prior to Bayesian phylogenetics. To investigate the virus diffusion in Spain, we reconstructed the virus diffusion history using an ancestral state reconstruction approach with a Bayesian stochastic search variable selection to determine the most probable spatial diffusion history. The Bayesian Skyride model was chosen to estimate the effective population size of H5N1 due to its suitability for single-locus data and its ability to infer smooth demographic trends without requiring user-defined time points (Minin et al. 2008, Minin and Suchard 2008, Gill et al. 2012). Temporal heterogeneity in sampling might introduce bias when using the Skyride model, thus the result derived from the model should be interpreted with caution.

The Hasegawa-Kishino-Yano (HKY) + Gamma nucleotide substitution model with an uncorrelated relaxed clock with lognormal distribution was used (Hasegawa et al. 1985). For discrete trait analysis (DTA), the asymmetric discrete trait substitution model was used to infer social network with the Bayesian stochastic search variable selection (BSSVS) and state change counts were reconstructed using Markov Jumps (Lemey et al. 2009). The Markov Chain Monte Carlo of the prepared configuration was run in parallel in three chains, each with 100 million steps and sampling every 10 000 steps. Log files produced from each chain were combined with 10% burn-in using the LogCombiner software v1.10.4. Combined chain log files were analysed with TRACER v1.7.1 (https://tree.bio.ed.ac.uk/software/tracer/) for > 200 effective sample size (Rambaut et al. 2018). Bayes factor (BF) was calculated using the SpreaD3 v0.9.7 (https://rega.kuleuven.be/cev/ecv/software/SpreaD3) from log files from the BSSVS analysis (Bielejec et al. 2016). Posterior probability of >0.5 and BF of >3 was identified as significant trait transitions (Kass and Raftery 1995). Maximum clade credibility (MCC) trees were generated using TreeAnnotator v1.10 and visualized using the Figtree 1.4.4 software. Actual migration rate was adjusted using the indicator data from rate log file.

For DTA, we assigned the discrete traits of the geographic region of Spain (Central: Madrid and Castilla_La_Mancha; North: Navarra, Cantabria, and Basque country; North-East: Catalonia and Aragon; North-West: Castilla_Leon, Asturias, and Galicia; South: Andalusia; and South-West: Extremadura) and outside of Spain (OOS). The phylodynamic analysis was also conducted to infer the diffusion dynamics between host types in Spain (captive bird, domestic chicken [which includes all production types, i.e. broilers, laying hens, breeders], domestic turkey, mink, wild Anseriformes, and wild non-Anseriformes) and all other viruses from OOS, thus providing grounds for which host type played a major role in the spread of H5N1 HPAI viruses in Spain. For a more detailed analysis of the most predominant genotype in Spain (Genotype EA-2021-AB), we assigned discrete traits of geographic region of Spain at the level of autonomous communities (Andalusia, Castilla_Leon, Castilla_La_Mancha, Catalonia, Extremadura, Madrid, and Navarra) and OOS. Wild birds, domestic poultry, and zoo birds were used as representative host discrete traits of Genotype EA-2021-AB viruses.

### Network analysis

To closely examine the genetic diversity within the most prevalent genotype (Genotype EA-2021-AB), we concatenated the all genome segments of each virus of Genotype EA-2021-AB. The concatenated linear coding region sequences were aligned using the MAFFT v7.453 software (Katoh and Standley 2013). Phylogenetic network analysis of the concatenated genomes of Genotype EA-2021-AB was performed using the median-joining method implemented in the program NETWORK v10 (https://www.fluxus-engineering.com/sharenet.htm) with epsilon set to 1 (Bandelt et al. 1999).

### Sampling bias

DTA of imbalanced dataset may lead to biased inference. The complete dataset and the subsampled dataset were tested for sample heterogeneity using tip trait randomization analysis (Edwards et al. 2011, Kwon et al. 2022, Veytsel et al. 2024). Ancestral root state frequencies were compared between the initial dataset and the subsampled dataset pre- and post-randomization. Correlation between trait sampling frequency and root state frequencies was examined. Trait transition estimated from Markov jump analysis was compared between the initial dataset and the subsampled dataset.

## Results

### Epidemiological data of H5N1 clade 2.3.4.4b HPAI outbreaks in Spain

A total of 124 complete genome sequences were generated and analysed (Supplementary Table S1). Among the 36 outbreaks of H5N1 clade 2.3.4.4b HPAI in poultry reported between December 2021 and November 2022 in Spain, chickens (*Gallus gallus*, *n* = 23 sequences) and turkeys (*Meleagris gallopavo*, *n* = 23 sequences) were both similarly affected. Of the 136 virus detections in wild birds, graylag geese (*Anser anser*, *n* = 22 sequences) and white storks (*Ciconia ciconia*, *n* = 12 sequences) were the most affected species. Andalusia (*n* = 45 sequences) and Castilla_Leon (*n* = 19 sequences) were the most impacted autonomous communities.

**Figure 1:**
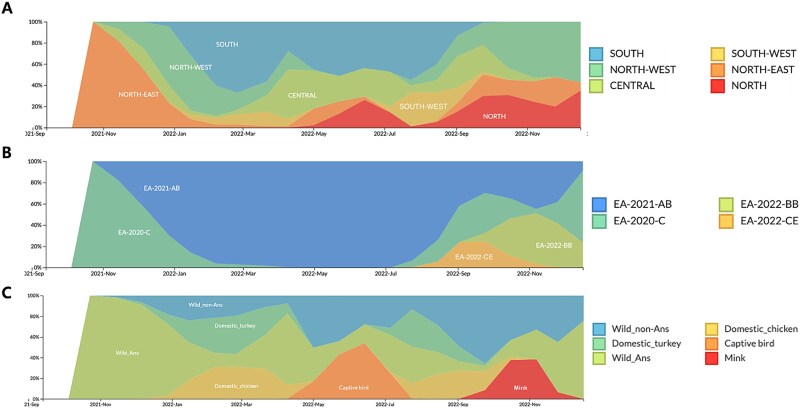
Frequency plot of (A) region, (B) genotype, and (C) host type of H5N1 clade 2.3.4.4b viruses in Spain from September 2021 to December 2022. *X*-axis represents time. *Y*-axis represents frequencies normalized to 100%. Colour legends are depicted at the panel on the right.

The H5N1 HPAI 2021–2022 epizootic in Spain consisted of two waves. The first wave reached its height in February 2022 (Supplementary Video 1). The peak of the second wave occurred during September 2022. Route of viral diffusion analysed with the Nextstrain build suggested multiple introductions of the viruses from other European countries into Spain between October 2021 and December 2022 (Supplementary Video 1). Initial outbreaks were reported in wild Anseriformes and then diversified into outbreaks in wild non-Anseriformes species, with subsequent detections in poultry (turkey and chicken farms), reaching the peak of the epizootic in February 2022 (Fig. 1C).

### Genetic diversity of H5N1 HPAI viruses in Spain

**Figure 2:**
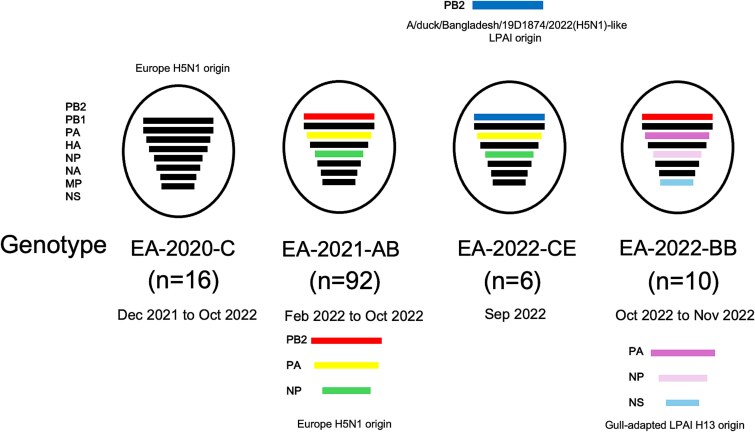
Graphical visualization of the genotypes identified of H5N1 clade 2.3.4.4b HPAI virus epizootic in Spain, 2021–2022. Each bar represents the eight segments of AI virus following the order on the left. Black gene segments are of European origin, while coloured gene segments illustrate reassortment events. The total number of sequences of each genotype is indicated in parentheses. The period indicated for each genotype refers to the dates of virus detection in Spain.

**Figure 3:**
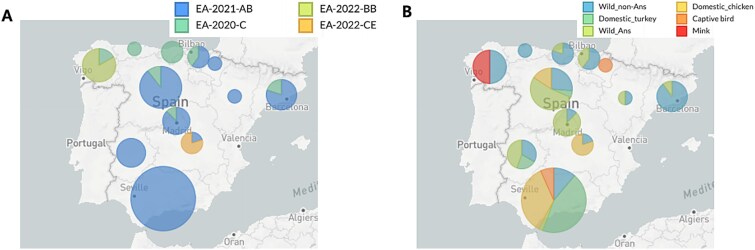
Pie chart map of (A) genotype and (B) host type of H5N1 clade 2.3.4.4b HPAI virus in each autonomous communities of Spain during the H5N1 clade 2.3.4.4b HPAI virus epizootic, September 2021 to December 2022. Pie sizes are proportionate to the number of sequences analysed in this study. Colour legends are depicted at the panel on the top-right.

The topology of ML trees, constructed using all eight AI virus genes, revealed a close relationship between Spain isolates and other European country isolates (Supplementary Fig. S1). The MCC tree of HA gene revealed two distinct HA subclades in Spain, Spain-1 and Spain-2, with subclade Spain-2 further dividing into subclades Spain-2.1, Spain-2.2, and Spain-2.2.1, assigned based on the MCC tree topology and the support on the node by posterior probability >0.9 (Supplementary Fig. S2). Genetic constellation of the H5N1 HPAI viruses from the 2021–2022 outbreaks in Spain were designated according to the phylogenetic analysis of all eight gene segments as Genotypes EA-2020-C (*n* = 16), EA-2021-AB (*n* = 94), EA-2022-CE (*n* = 6), and EA-2022-BB (*n* = 10), following the previously described genotype nomenclature (Figs 1B and 2) (Fusaro et al. 2024). The genome constellation of Genotype EA-2020-C is constituted by European H5N1 clade 2.3.4.4b HPAI virus-origin internal genes possessing HA subclade Spain-1, affecting northern regions of Spain. Genotype EA-2021-AB comprises most of the viruses detected in the 2021–2022 Spanish outbreaks, affecting most regions in Spain (Fig. 1A and B). Genotype EA-2021-AB, which contains internal gene segments of a recent 2022 European H5N1 HPAI virus, is further characterized into two distinct clusters namely AB1, possessing HA subclade Spain-2.1, and AB2, possessing HA subclade Spain-2.2 (Fig. 2 and Supplementary Fig. S2). Genotype EA-2022-CE viruses are the result of a reassortment of Genotype EA-2021-AB viruses with LPAI virus-origin polymerase basic protein 2 (PB2) gene. Genotype EA-2022-CE possessed HA subclade Spain-2.2 and resulted in an outbreak in laying hens in Castilla_La_Mancha (Figs 1 and 3). The genome constellation of Genotype EA-2022-BB, previously reported as “A/gull/France/22P015977/2022-like genotype,” consists of European H5N1 clade 2.3.4.4b HPAI virus backbone with gull-adapted LPAI virus H13-origin polymerase acidic, nucleoprotein, and non-structural genes (Agüero et al. 2023, Fusaro et al. 2024). Genotype EA-2022-BB possessed HA subclade Spain-2.2.1 and caused an outbreak that was limited to Galicia in Spain, involving a mink farm (Figs 1A and 3A).

### Evolutionary origin of H5N1 HPAI virus genotypes in Spain

The time to the most recent common ancestor (tMRCA) was calculated using the HA segment of the H5N1 HPAI virus genotypes described here to study their evolutionary origins and emergence (Supplementary Fig. S2). Genotype EA-2020-C viruses carrying Spain-1 HA gene were most likely introduced into Spain on multiple occasions originating from the most recent common ancestor which emerged around 27 July 2021 (95% highest posterior density (HPD): 4 July 2021 to 6 September 2021). The introduction of cluster Spain-2.1 viruses into Spain was estimated to have occurred around 7 December 2021 (95% HPD: 5 November 2021 to 4 January 2022). The emergence of Genotype EA-2021-AB of cluster Spain-2.2 viruses was estimated around 26 August 2021 (95% HPD: 18 July 2021 to 30 September 2021). Genotype EA-2022-CE of cluster Spain-2.2 viruses was estimated to have emerged around 10 August 2022 (95% HPD: 8 July 2022 to 5 September 2022). Introduction of cluster Spain-2.2.1 viruses into Spain was estimated around 4 August 2022 (95% HPD: 30 June 2022 to 24 September 2022) (Supplementary Fig. S2).

### Geographic spread of H5N1 clade 2.3.4.4b HPAI viruses into Spain

**Figure 4:**
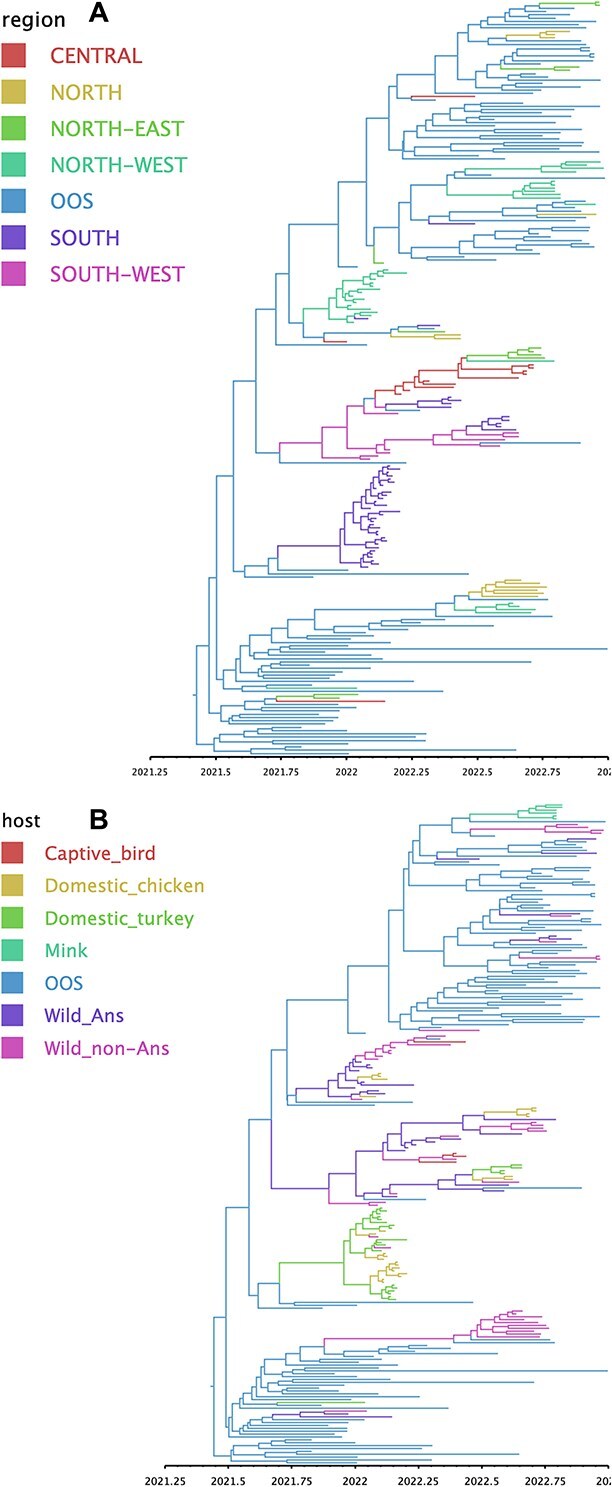
(A) Bayesian phylogenetic tree of HA gene of clade 2.3.4.4.b H5N1 HPAI viruses, where region traits were defined in the analysis as discrete states. The region traits were designated as “Central,” “North,” ““North-East,” “North-West,” “South,” “South-West,” and “outside of Spain” (OOS). (B) Bayesian phylogenetic tree of HA gene of H5N1 clade 2.3.4.4b HPAI viruses, where host traits were defined in the analysis as discrete states. The host traits were designated as “captive birds,” “domestic chicken,” “domestic Turkey,” “mink,” “wild Anseriformes,” “wild non-Anseriformes,” and “OOS” (isolates from outside of Spain regardless of the host type). Posterior probabilities of nodes are indicated by the size of the circles at each node. The colour representation of the branches is indicated by the legend in the top-left of each panel.

**Figure 5:**
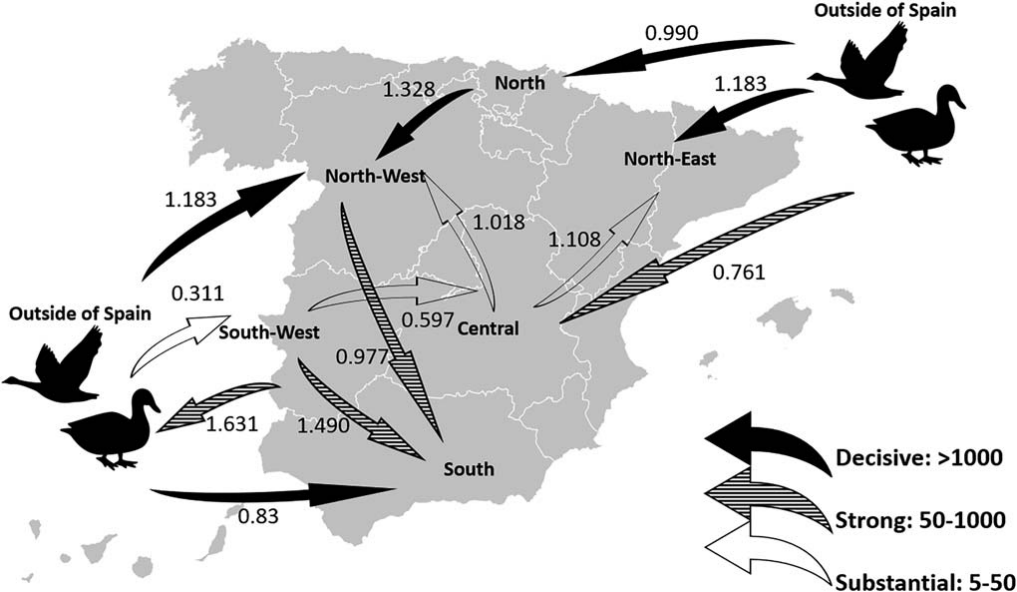
Inferred transmission networks of H5N1 clade 2.3.4.4b HPAI viruses among geographic location of Spain and viruses originating from outside of Spain. Direction of the diffusion is indicated by the direction of the arrows. The arrows indicate well supported bayes factor (BF); solid arrows indicate statistical support with BF > 100 (decisive), striped arrows indicate support with 10 < BF < 100 (strong), and open arrows indicate support with 3 < BF < 10 (substantial).

To reconstruct spatial and temporal viral diffusion into and within Spain, we conducted a DTA of H5N1 clade 2.3.4.4b HPAI viruses. The DTA revealed multiple and repeated introductions of H5N1 clade 2.3.4.4b HPAI viruses circulating in wild birds in Europe. The actual viral migration rate among the geographic regions revealed that viruses circulating in Europe (OOS) were introduced to each geographical region of Spain (North, North-East, North-West, Central, South, and South-West) at least once throughout 2021–2022 (Figs 4A and 5 and Supplementary Video 1).

**Figure 6:**
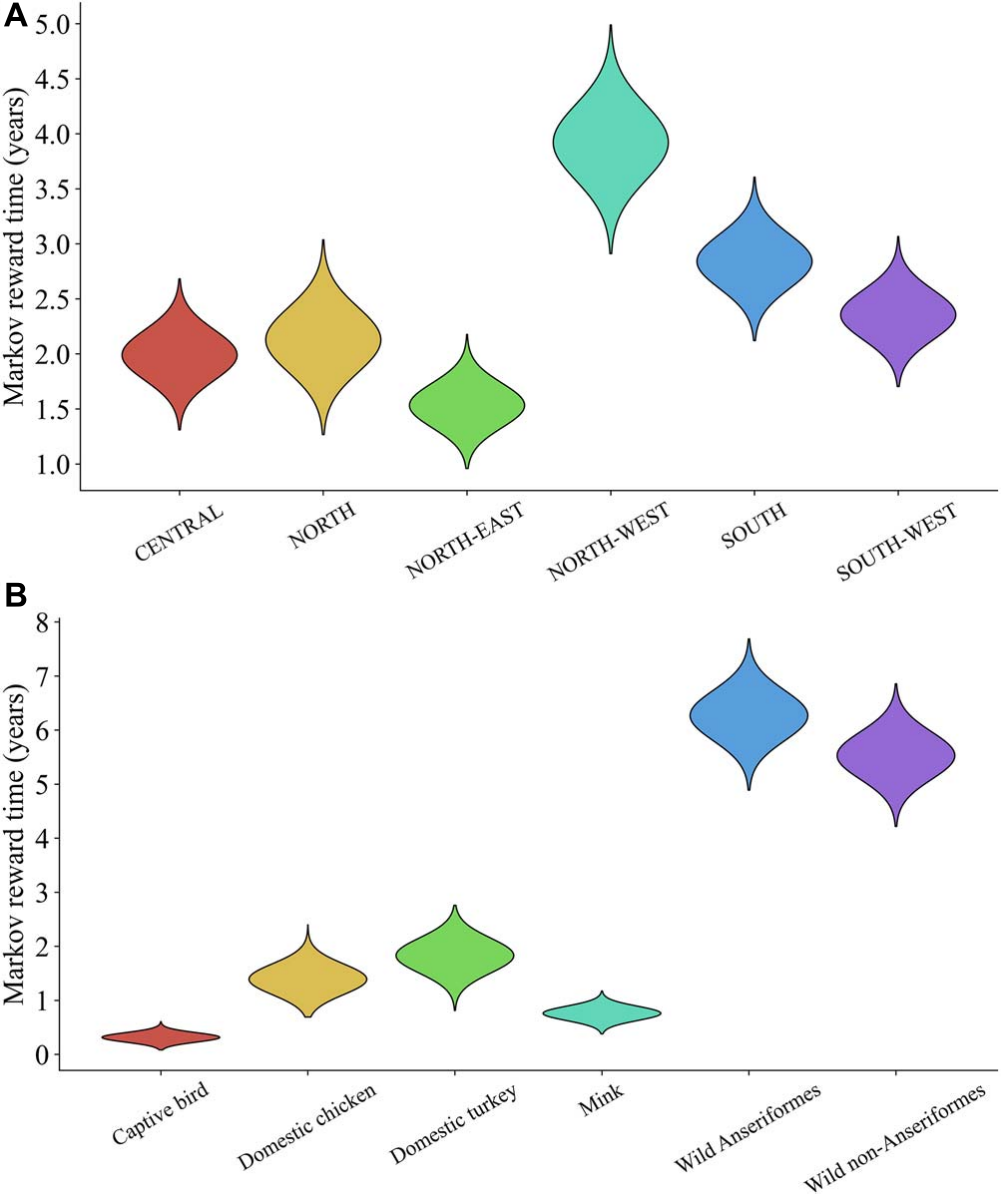
Markov reward times for discrete traits (*X*-axis) of H5N1 clade 2.3.4.4b HPAI viruses depicted using violin plots. Density distribution of the total time spent represented by Markov reward time in years (*Y*-axis) for the discrete traits of (A) geographic locations and (B) host types in Spain.

Within Spain, viral movement from the South-West of Spain to the Central and Southern regions was observed with high Bayesian support (Fig. 5 and Table 1). Viral movement from the Central region of Spain to the North-Western and North-Eastern regions was observed with substantial Bayesian support. Viral migration from the South-West, North-West, and OOS to the South of Spain was also highly supported (Table 1). The Markov reward times represent the cumulative evolutionary time (in years) the virus lineage spent in each discrete trait across the trees. The Markov reward times among the geographic regions of Spain were highest in the North-West (3.922; 95% HPD: 2.911–4.989), with South (2.841; 95% HPD: 2.121–3.605) and South-West (2.355; 95% HPD: 1.704–3.067) regions following closely (Fig. 6A).

**Table 1:** Transition rate, bayes factor, and posterior probability (>0.5) of discrete trait phylodynamic analysis between geographic locations in Spain and outside of Spain of H5N1 viruses in Spain

Transition from	Transition to	Mean actual migration rate^a^ [95% BCI]^b^	Bayes Factor	Posterior probability
OOS^c^	NORTH-WEST	1.1834 [0.1878, 2.4665]	6325.518	0.999
OOS	NORTH	0.9897 [0.1573, 2.0959]	1486.273	0.996
OOS	NORTH-EAST	1.0675 [0.1429, 2.2771]	1125.224	0.995
OOS	SOUTH	0.83 [0.078, 1.842]	1065.724	0.995
SOUTH-WEST	OOS	1.6312 [0, 3.5675]	461.751	0.989
SOUTH-WEST	SOUTH	1.4903 [0, 3.3858]	361.373	0.986
NORTH-WEST	SOUTH	0.9767 [0, 2.4558]	94.825	0.947
OOS	CENTRAL	0.7612 [0, 1.7484]	65.084	0.925
CENTRAL	NORTH-EAST	1.1082 [0, 2.8411]	52.162	0.908
CENTRAL	NORTH-WEST	1.0184 [0, 2.7264]	36.031	0.872
NORTH	NORTH-WEST	1.3283 [0, 3.4766]	21.115	0.8
OOS	SOUTH-WEST	0.3108 [0, 0.9537]	15.757	0.749
SOUTH-WEST	CENTRAL	0.5966 [0, 2.1742]	7.263	0.579

a Actual migration rates were calculated by multiplying rate and indicator.

b BCI: Bayesian credibility interval.

c OOS: Outside of Spain.

**Figure 7:**
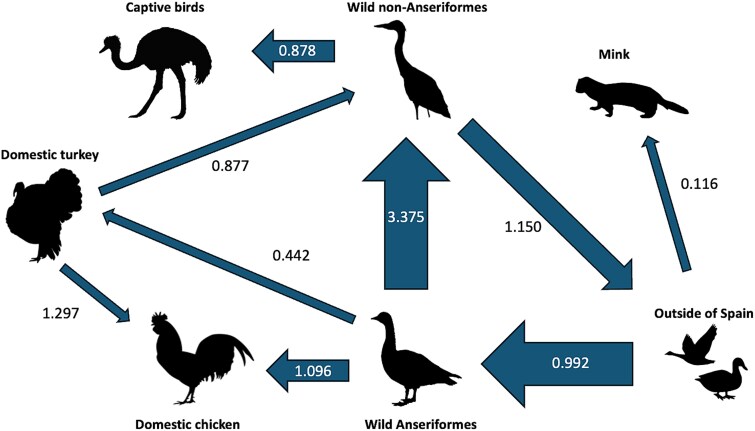
Inferred diffusion networks of H5N1 clade 2.3.4.4b HPAI viruses among host types of Spain and viruses originating from outside of Spain. Direction of the diffusion is indicated by the direction of the arrows. The width of the arrows is scaled by well-supported bayes factor (posterior probability > 0.5). The number at each arrow indicates the adjusted migration rate of each diffusion event.

Viral migration rate between host types indicated strong support for diffusion from OOS to wild Anseriformes in Spain (BF: 282032, posterior probability (PP): 1.00), and from wild Anseriformes to wild non-Anseriformes within Spain (BF: 282032, PP: 1.00) (Fig. 7 and Table 2). Wild Anseriformes also contributed to the diffusion of viruses to domestic chickens (BF: 366, PP: 0.99) and domestic turkeys (BF: 8, PP: 0.61). Viral migration from wild non-Anseriformes in Spain to OOS was also highly supported (BF: 727, PP: 0.99), indicating virus movement to other European countries (Table 2).

**Table 2:** Transition rate, bayes factor, and posterior probability (>0.5) of discrete trait phylodynamic analysis between host types of H5N1 viruses in Spain

Transition from	Transition to	Mean actual migration rate^a^ [95% BCI]^b^	Bayes factor	Posterior probability
OOS^c^	Wild_Anseriformes	0.9921 [0.2631, 1.8832]	282031.88	1.00
Wild_Anseriformes	Wild_non-Anseriformes	3.3753 [1.1074, 6.0805]	282031.88	1.00
Wild_non-Anseriformes	OOS	1.1502 [0, 2.4446]	727.29	0.99
Wild_Anseriformes	Domestic_chicken	1.0957 [0, 2.3563]	365.83	0.99
Wild_non-Anseriformes	Captive_bird	0.8777 [0, 1.9516]	355.39	0.99
Domestic_turkey	Wild_non-Anseriformes	0.877 [0, 2.3156]	22.11	0.81
Domestic_turkey	Domestic_chicken	1.2965 [0, 3.3287]	14.13	0.73
OOS	Mink	0.116 [0, 0.3809]	13.35	0.72
Wild_Anseriformes	Domestic_turkey	0.4423 [0, 1.5284]	8.21	0.61

a Actual migration rates were calculated by multiplying rate and indicator.

b BCI: Bayesian credibility interval.

c OOS: Outside of Spain.

Markov reward time of host types in Spain was highest in wild birds such as wild Anseriformes (6.271; 95% HPD: 4.891–7.688) and wild non-Anseriformes (5.530; 95% HPD: 4.219–6.856) (Fig. 6B). Poultry species such as domestic turkey (1.829; 95% HPD: 0.814–2.759) and domestic chicken (1.391; 95% HPD: 0.693–2.397) also contributed, albeit to a lesser extent, to the circulation and maintenance of virus in Spain.

### Geographic spread of H5N1 HPAI genotype EA-2021-AB viruses circulating in Spain

We conducted a DTA of the most predominant Genotype EA-2021-AB using autonomous regions to further understand the spatial and temporal diffusion of these viruses. The DTA revealed that Genotype EA-2021-AB viruses circulating in wild birds in Europe were introduced into the wild bird populations of Castilla_Leon and Extremadura (Supplementary Figs S3A and S4). Introduction of Genotype EA-2021-AB viruses into wild birds of Castilla_Leon was estimated around 24 November 2021 (95% HPD: 13 October 2021 to 29 December 2021). The incursion of these viruses into wild birds of Extremadura was estimated around 24 December 2021 (95% HPD: 19 November 2021 to 23 January 2022) (Supplementary Fig. S3A). Viral diffusion from wild bird populations OOS to wild bird populations of Extremadura and Castilla_Leon was statistically significant (Supplementary Fig. S4 and Supplementary Table S2).

The Markov reward times (denoting time in years spent in the region) of Genotype EA-2021-AB among the autonomous communities of Spain were highest in Andalusia (2.533; 95% HPD: 1.712–3.406), Castilla_Leon (1.667; 95% HPD: 0.958–2.435), and Extremadura (1.965; 95% HPD: 1.212–3.177) (Supplementary Fig. S5A). The actual viral migration rate among the autonomous communities revealed that Castilla_Leon and Extremadura were the main virus donor regions during the 2021–2022 Spain H5N1 HPAI epizootic (Supplementary Fig. S5A). The BSSVS analysis of diffusion between geographic regions is in line with the MCC tree topology; virus movement from Extremadura and Castilla_Leon to Andalusia was highly supported (BF: 621.711 and 147.584, PP: 0.99 and 0.96, respectively) (Supplementary Fig. S4 and Supplementary Table S2).

### Host species contributing to H5N1 HPAI genotype EA-2021-AB virus spread within Spain

To reconstruct spatial and temporal viral diffusion of Genotype EA-2021-AB among different hosts, we conducted a DTA using host types (domestic poultry, wild birds, and captive zoo birds). The DTA revealed that there were three major viral introductions of genotype EA-2021-AB from wild birds to poultry and two introductions from wild birds to zoo birds (Supplementary Fig. S3B). The transition from wild birds to domestic poultry was strongly supported (BF: 55259.365, PP: 1.00) (Supplementary Table S3). The highest total Markov reward time among host types was in wild birds (5.491; 95% HPD: 4.136–7.043), followed by domestic poultry (1.805; 95% HPD: 1.229–2.458) and captive zoo birds (0.421; 95% HPD: 0.222–0.635) (Supplementary Fig. S5B). The actual viral migration rate between host types suggests that wild birds were the major virus donor hosts to both domestic poultry and captive birds (Supplementary Fig. S6 and Supplementary Table S3).

### Network analysis of H5N1 HPAI genotype EA-2021-AB viruses in Spain

**Figure 8:**
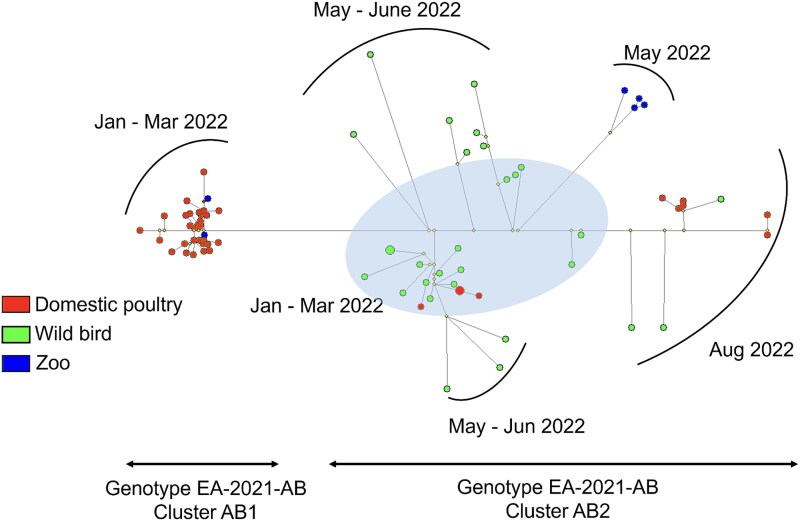
Network tree of genotype EA-2021-AB generated using the median-joining method on NETWORK 10.2.0.0 (https://www.fluxus-engineering.com). Red circles represent viruses from domestic poultry. Green circles represent viruses from wild birds. Blue circles represent viruses from zoo birds. Yellow circles represent median vector nodes. The blue shaded area represents viruses that were isolated from January 2022 through march 2022. Brackets indicate the time frame of detection of the isolates. Branch lengths are proportionate to the number of mutations, and the node sizes represent frequency.

In line with the phylogenetic analysis, the network analysis supported that Genotype EA-2021-AB viruses are divided into clusters AB1 and AB2 (Fig. 8). On the one hand, cluster AB1 viruses were mostly isolated from domestic poultry outbreaks during a relatively short time spanning from January 2022 to March 2022 in Andalusia. These cluster AB1 viruses are closely related to each other on the network tree, suggesting low genetic diversity among them. On the other hand, cluster AB2 consists of mostly wild bird isolates and sporadic outbreaks of domestic poultry and zoo bird outbreaks maintained for a longer time from January 2022 to August 2022 in multiple regions of Spain (Fig. 8). Cluster AB2 viruses were more dispersed on the network tree, suggesting higher genetic diversity among them.

### Sampling bias

The root state probability was highly correlated to the number of trait taxa in the complete dataset (*n* = 231) (Supplementary Fig. S7). The complete dataset was subsampled to have balanced host (*n* = 137) and location (*n* = 119) traits (Supplementary Tables S4 and S5). Root state probability of the subsampled dataset was roughly balanced throughout similar count traits (Supplementary Tables S4 and S5). Markov transition of host and location trait of the balanced dataset were compared to that of the complete dataset, which showed overlapping transition events. Five out of seven transitions in the subsampled host dataset were overlapping with the transition events observed in the complete dataset (Supplementary Table S6). All 10 transitions in the subsampled location dataset were also observed in the transition events from the complete dataset (Supplementary Table S7).

## Discussion

The propensity for H5Nx clade 2.3.4.4b viruses to reassort and create novel genome constellations is unprecedented since their most recent emergence (Lee et al. 2017, Xie et al. 2023), and the latest surge in mammalian H5 HPAI virus infections are concerning for wildlife and public health (EFSA et al. 2022a, 2023b). Thus, generating and publicly sharing complete genome viral sequences from infected wild birds and poultry in a timely fashion ensures an optimal monitoring of circulating viruses and rapid understanding of viral evolution and associated risks (Agüero et al. 2023, Aznar et al. 2023, EFSA et al. 2023b). Here, we identified the origins and the evolutionary and diffusion dynamics of the H5N1 HPAI viruses sampled during the 2021–2022 epizootic in Spain, the worst AI season ever recorded in the country.

Bayesian phylogenetic analysis of the HA gene suggested multiple wild bird introductions of H5N1 clade 2.3.4.4b HPAI viruses into Spain from other European countries. In fact, we revealed that all the affected Spanish regions had experienced direct incursion of the virus from other European countries. In turn, the South-Western region and wild non-Anseriformes acted as virus donors to other countries in Europe. This indiscriminating virus exchange pattern among different European countries and host types further obfuscates our ability to understand viral movement and evolution (Fusaro et al. 2024).

Genetic characterization of whole genome sequences revealed the circulation of four distinct genotypes (EA-2020-C, EA-2021-AB, EA-2022-CE, and EA-2022-BB) of H5N1 clade 2.3.4.4b HPAI viruses in Spain during the 2021–2022 season. Genotypes EA-2020-C, EA-2021-AB, and EA-2022-BB have been reported in other countries in Europe prior to their detection in Spain, while Genotype EA-2022-CE was found unique to Spain, resulting from reassortment with co-circulating LPAI viruses. Similarly, 2016–2017, 2020–2021, and 2021–2022 H5Nx HPAI clade 2.3.4.4b epizootics in other European countries were characterized by the emergence and circulation of multiple reassortants and distinct genotypes (Briand et al. 2022, Engelsma et al. 2022, King et al. 2022a, 2022b, Nagy et al. 2023, Xie et al. 2023, Fusaro et al. 2024).

Here, the most prevalent was Genotype EA-2021-AB, with cluster AB1 viruses mostly linked to poultry outbreaks involving lateral spread among farms, while cluster AB2 viruses circulated primarily in wild birds, but also poultry and captive birds (Fig. 8). The minimal mutations between each poultry isolate in the cluster AB1 are shown by the shorter branches between the nodes, suggesting inter-farm transmissions. The long branches seen in cluster AB2 viruses suggest more virus genetic diversity in this population, with evolution over time contributing to the genetic diversity. It should be noted that the median-joining network tree does not reliably infer the root and the directionality of the mutations.

Genotype EA-2022-CE viruses are reassortants of Genotype EA-2021-AB viruses with PB2 of wild bird LPAI virus-origin. We could only speculate that the reassortment most likely occurred in wild birds and was introduced to poultry as a single outbreak, rather than the reassortment occurring in poultry, based on the existing phylogenetic evidence and the absence of further detection of this reassortant genotype in other wild or poultry birds (Fusaro et al. 2024). The ML tree of the PB2 gene indicated that the PB2 gene of genotype EA-2022-CE clustered with the isolates from Bangladesh, which was not observed in other internal genes (Supplementary Fig. S1A). The origin of the Bangladesh PB2 gene remains uncertain and may be due to multiple reassortment events or unsampled intermediates along migratory routes. Surveillance gaps must be addressed to allow for more accurate inference of the origin of this genotype.

Genotype EA-2022-BB viruses are reassortants with gull-adapted H13 LPAI virus-origin. This genotype was associated with the expanding host range of H5N1 HPAI viruses and their wide circulation in colony-breeding seabirds during the summer of 2022 (Agüero et al. 2023, EFSA et al. 2023b). Indeed, this genotype was extensively detected in seabirds in the Netherlands, Belgium, and France during the summer months (EFSA et al. 2023b) and includes the H5N1 HPAI virus that later infected farmed minks in Galicia, Spain in October 2022 (Agüero et al. 2023). The mink outbreak stirred up concerns as further investigations indicated apparent onward mink-to-mink viral transmission within the affected farm, suggesting these viruses may gain mutations advantageous to mammalian infection (Agüero et al. 2023). Concerningly, since then, H5N1 clade 2.3.4.4b HPAI virus infections in mammals have been detected at an alarming rate all around the world especially in Europe, North America, and South America (Alkie et al. 2022, Leguia et al. 2023, de Carvalho Araujo et al. 2024, Plaza et al. 2024).

Tip trait randomization initially suggested that the complete dataset used for the Bayesian phylodynamic analysis (*n* = 231) was biased toward the most frequent host and location traits, OOS (Supplementary Tables S4 and S5). Subsampling of the original dataset improved the root state probability to be balanced throughout the traits, with low correlation between the number of traits counts and root state probability (Supplementary Figs S7 and S8). The Markov transitions of the complete and subsampled datasets were substantially overlapping, producing similar results (Supplementary Tables S6 and S7).

Evidence from phylogeny, diffusion networks, and Bayesian phylodynamic analysis of H5N1 clade 2.3.4.4b HPAI viruses support that the initial entry of the virus into Spain was in Castilla Leon and Extremadura. The subsequent spread of the virus to Madrid, Andalusia, and Catalonia most likely was a combination of viral diffusion among wild birds within Spain and separate virus introduction from wild birds OOS, suggesting a scenario in which both types of incursions coexisted. Frequent incursion and exchange in these regions may be partly due to major wetlands like Doñana National Park in south of Spain, a major stopover site along the migratory route between Europe and Africa. Introductions directly from Africa to the Iberian Peninsula were not supported in our phylogenetic analyses and in other studies with a more holistic approach (Xie et al. 2023, Fusaro et al. 2024). These five autonomous communities include municipalities that are ranked as very high and/or high for risk of introduction of AI through migratory wild birds based on the census of waterfowl in wetlands, HPAI outbreaks in Europe and migratory bird movements, weather conditions that impact survival of the virus, density of poultry farms, and trade movements of poultry from the EU and within the country (Garrido 2023). Andalusia appears to have had introductions of cluster AB viruses in five separate instances; of those, two were limited to a single case, while the other three were maintained within the region for some time. The virus was detected in Andalusian wild birds after the first gallinaceous poultry outbreak, presumably because of post-outbreak surveillance, although a spillback event cannot be excluded. In line with other clade 2.3.4.4b European outbreaks (Guinat et al. 2022, King et al. 2022a, Nagy et al. 2023), we can hypothesize that the viruses had already been circulating in wild birds in Andalusia for a while and subsequently transmitted to poultry. In fact, epidemiological investigation of poultry outbreaks suggests that contact with wild birds was the most likely route of introduction of the virus into poultry premises (Influenza aviar 2018). The first detection in Andalusian poultry in January 2022 led to the biggest poultry outbreak of the season in Spain, which lasted until March 2022 and involved lateral farm-to-farm spread among turkey and chicken premises (Influenza aviar 2018). Upon containment of this initial outbreak, a few more outbreaks were detected in this region, notably in the Cordoba Zoo in late May to June 2022 and in two poultry (chicken and turkey) farms in August 2022. The virus isolated from these poultry outbreaks was closely related to the wild graylag goose virus (A/*A. anser*/Spain/2825–1 22VIR8632-13/2022) isolated in Extremadura, a neighbouring autonomous community. It should be noted that the estimated tMRCAs represent the most recent divergence among sampled Spanish viruses and thus serve as a baseline estimate of the introduction window. The actual introduction may have occurred earlier, along the ancestral branch leading to the Spanish clade, within the broader HPD interval, and the immediate ancestor may or may not have been detected or reported.

Our results revealed intricate viral diffusion dynamics between different host types. After the initial entry of the virus into wild Anseriformes in Spain, viral diffusion from wild Anseriformes to wild non-Anseriformes, domestic chicken, and domestic turkey contributed to viral spread throughout Spain. This highlights the major role of wild birds in the maintenance and dissemination of H5N1 clade 2.3.4.4b HPAI viruses during the 2021–2022 epizootic.

The largest proportion of wild bird cases during the 2021–2022 season in Spain occurred in graylag geese and white storks, which are also featured among the most affected species in the European 2021–2022 epizootic in general and on the Iberian Peninsula in particular (Authority et al. 2022, EFSA et al. 2022a, 2022b). The role of these two species in the epidemiology of HPAI on the Iberian Peninsula remains to be elucidated. However, geese are a long-known reservoir for AI viruses worldwide and can suffer fatal clade 2.3.4.4b HPAI infections (Graziosi et al. 2024). Also, storks are known to harbour LPAI viruses in Southern Europe (Pérez-Ramírez et al. 2010, Bárbara et al. 2017) and are highly susceptible to H5N8 clade 2.3.4.4b HPAI infection (Globig et al. 2017). It is worth highlighting that Spain, as many other European countries, relies on passive surveillance for AI in wild birds. Therefore, we cannot exclude that unsampled populations or species played a vital role in viral maintenance and diffusion (Xie et al. 2023, Andrew et al. 2024, Kim et al. 2024).

Our results show that wild birds played a key role in maintaining H5N1 HPAI viruses in Spain and were the most likely source of virus incursion to domestic poultry and zoo birds. The implication of wild birds in the maintenance of clade 2.3.4.4b HPAI viruses, subsequently yielding increasing opportunities for spillover to poultry, has been widely documented (Lycett et al. 2020, Xie et al. 2023, Kim et al. 2024). In our study, Andalusia (South) was the hotspot for maintenance of viruses in commercial flocks through domestic poultry networks and associated human activity, while viruses in Castilla_Leon (North-West) and Extremadura (South-West) were maintained largely due to circulation in wild birds.

## Conclusions

Our phylodynamic analyses indicated multiple introductions of H5N1 clade 2.3.4.4b HPAI viruses in Spain during the 2021–2022 season. The H5N1 viruses rapidly evolved through reassortment and extensive circulation in wild birds. Enhanced and coordinated sequencing efforts and molecular phylodynamic analysis were able to provide a more holistic view of the epidemiology and ecology of clade H5N1 2.3.4.4b HPAI viruses. The findings from this study will be useful for policy making and future HPAI virus outbreak response.

## Supplementary Material

supplementary-material_veag006

## Data Availability

The data supporting the findings of this study are available in Global Initiative on Sharing All Influenza Data (Supplementary Table S8).
