# Supplementary material for: Wild birds drive the introduction, maintenance, and spread of H5N1 clade 2.3.4.4b high pathogenicity avian influenza viruses in Spain, 2021–2022
Source: Virus Evol. 2026 Jan 30;12(1):veag006. doi: 10.1093/ve/veag006 (PMC12931561; doi:10.1093/ve/veag006)
Supplement: supplementary-material_veag006 [file supplementary-material_veag006.zip › Supplementary_Table_S2_KBD_veag006.docx]

Table S2. Transition rate, Bayes factor, and posterior probability (>0.5) of discrete trait phylodynamic analysis between autonomous communities of H5N1 HPAI Genotype EA-2021-AB viruses in Spain and outside of Spain

| Transition from | Transition to | Mean actual migration rate^a^ [95% BCI]^b^ | Bayes Factor | Posterior probability |
| --- | --- | --- | --- | --- |
| Extremadura | Andalusia | 1.6621 [0, 3.5531] | 621.711 | 0.99 |
| Castilla_Leon | Andalusia | 1.6654 [0, 3.7806] | 147.584 | 0.96 |
| Extremadura | Madrid | 0.9817 [0, 2.5248] | 76.110 | 0.92 |
| Madrid | OOS^c^ | 1.1438 [0, 2.9516] | 52.677 | 0.89 |
| OOS | Castilla_Leon | 0.5401 [0, 1.6623] | 18.703 | 0.75 |
| Extremadura | OOS | 1.1769 [0, 3.5847] | 15.905 | 0.72 |
| OOS | Extremadura | 0.5185 [0, 1.6763] | 15.293 | 0.71 |
| Castilla_Leon | Catalonia | 0.9283 [0, 2.8418] | 13.796 | 0.69 |
| Catalonia | Navarra | 0.5983 [0, 2.4091] | 6.906 | 0.52 |
| Andalusia | Castilla_La_Mancha | 0.3963 [0, 1.6001] | 6.292 | 0.50 |

^a^ Actual migration rates were calculated by multiplying rate and indicator.

^b^ BCI: Bayesian credibility interval.

^c^ OOS: Outside of Spain.
