# Supplementary material for: Wild birds drive the introduction, maintenance, and spread of H5N1 clade 2.3.4.4b high pathogenicity avian influenza viruses in Spain, 2021–2022
Source: Virus Evol. 2026 Jan 30;12(1):veag006. doi: 10.1093/ve/veag006 (PMC12931561; doi:10.1093/ve/veag006)
Supplement: supplementary-material_veag006 [file supplementary-material_veag006.zip › Supplementary_Table_S3_KBD_veag006.docx]

Supplementary Table S3. Transition rate, Bayes factor, and posterior probability (>0.5) of discrete trait phylodynamic analysis between host types of H5N1 HPAI Genotype EA-2021-AB viruses in Spain

| Transition from | Transition to | Mean actual migration rate^a^ [95% BCI]^b^ | Bayes Factor | Posterior probability |
| --- | --- | --- | --- | --- |
| Wild | Domestic | 1.7051 [0.1652, 3.7436] | 55259.365 | 1.00 |
| Wild | Zoo | 0.8073 [0, 2.0127] | 179.362 | 0.99 |
| Domestic | Wild | 1.178 [0, 2.8193] | 132.900 | 0.99 |

^a^ Actual migration rates were calculated by multiplying rate and indicator.

^b^ BCI: Bayesian credibility interval.
