# Supplementary material for: Wild birds drive the introduction, maintenance, and spread of H5N1 clade 2.3.4.4b high pathogenicity avian influenza viruses in Spain, 2021–2022
Source: Virus Evol. 2026 Jan 30;12(1):veag006. doi: 10.1093/ve/veag006 (PMC12931561; doi:10.1093/ve/veag006)
Supplement: supplementary-material_veag006 [file supplementary-material_veag006.zip › Supplementary_Table_S4_new_KBD_veag006.docx]

**Supplementary Table S4. Tip trait randomization of location trait.** Number of sequences and root location state frequency (%) of each discrete state

**^a^ OOS: Outside of Spain.**

|  | Complete dataset (n=231) | | Down-sampled dataset (n=119) | |
| --- | --- | --- | --- | --- |
| Discrete state | Number of sequences (%) | Root location state frequency | Number of sequences (%) | Root location state frequency |
| SOUTH-WEST | 9 (3.90%) | 0.86% | 9 (7.89%) | 2.83% |
| NORTH-EAST | 12 (5.19%) | 0.02% | 12 (10.53%) | 2.50% |
| NORTH | 11 (4.76%) | 0.08% | 11 (9.65%) | 8.20% |
| CENTRAL | 13 (5.63%) | 0.02% | 13 (11.40%) | 14.10% |
| SOUTH | 45 (19.48%) | 0.03% | 23 (20.18%) | 21.41% |
| NORTH-WEST | 33 (14.29%) | 0.02% | 23 (20.18%) | 39.60% |
| **OOS^a^** | 108 (46.75%) | 98.98% | 28 (24.56%) | 11.35% |
