# Supplementary material for: Wild birds drive the introduction, maintenance, and spread of H5N1 clade 2.3.4.4b high pathogenicity avian influenza viruses in Spain, 2021–2022
Source: Virus Evol. 2026 Jan 30;12(1):veag006. doi: 10.1093/ve/veag006 (PMC12931561; doi:10.1093/ve/veag006)
Supplement: supplementary-material_veag006 [file supplementary-material_veag006.zip › Supplementary_Table_S5_new_KBD_veag006.docx]

**Supplementary Table S5. Tip trait randomization of location trait data.** Number of sequences and root location state frequency (%) of each discrete state

|  | Complete dataset (n=231) | | Down-sampled dataset (n=137) | |
| --- | --- | --- | --- | --- |
| Discrete state | Number of sequences (%) | Root location state frequency | Number of sequences (%) | Root location state frequency |
| Captive_bird | 4 (1.73%) | 0.04% | 4 (2.92%) | 2.83% |
| Domestic_chicken | 23 (9.96%) | 0.04% | 23 (16.79%) | 29.39% |
| Domestic_turkey | 23 (9.96%) | 0.02% | 23 (16.79%) | 28.01% |
| Wild_Ans | 27 (11.69%) | 0.22% | 27 (19.71%) | 18.67% |
| Mink | 6 (2.60%) | 0.04% | 6 (4.38%) | 1.39% |
| OOS^a^ | 108 (46.75%) | 98.36% | 28 (20.44%) | 25.18% |
| Wild_non-Ans | 40 (17.32%) | 1.29% | 26 (18.98%) | 4.53% |

^a^ OOS: Outside of Spain.
