# Supplementary material for: Wild birds drive the introduction, maintenance, and spread of H5N1 clade 2.3.4.4b high pathogenicity avian influenza viruses in Spain, 2021–2022
Source: Virus Evol. 2026 Jan 30;12(1):veag006. doi: 10.1093/ve/veag006 (PMC12931561; doi:10.1093/ve/veag006)
Supplement: supplementary-material_veag006 [file supplementary-material_veag006.zip › Supplementary_Table_S6_new_KBD_veag006.docx]

**Supplementary Table S6. Transmission matrix of Bayesian stochastic search variable selection analysis of host trait.** Overlapping transitions are shown in bold

| Dataset | From | To | Bayes factor | Posterior Probability |
| --- | --- | --- | --- | --- |
| Complete dataset (n=231) | **OOOS**^a^ | **Wild_Ans** | **282031.88** | **1.00** |
|  | **Wild_Ans** | **Wild_non-Ans** | **282031.88** | **1.00** |
|  | Wild_non-Ans | OOS | 727.29 | 0.99 |
|  | **Wild_Ans** | **Domestic_chicken** | **365.83** | **0.99** |
|  | **Wild_non-Ans** | **Captive_bird** | **355.39** | **0.99** |
|  | **Domestic_turkey** | **Wild_non-Ans** | **22.11** | **0.81** |
|  | Domestic_turkey | Domestic_chicken | 14.13 | 0.73 |
|  | OOS | Mink | 13.35 | 0.72 |
|  | Wild_Ans | Domestic_turkey | 8.21 | 0.61 |
| Down-sampled dataset (n=137) | **Wild_Ans** | **Wild_non-Ans** | **237399.47** | **1.00** |
|  | OOS | **Wild_Ans** | **3160.12** | **1.00** |
|  | **Wild_non-Ans** | **Captive_bird** | **447.79** | **0.99** |
|  | **Wild_Ans** | **Domestic_chicken** | **396.43** | **0.99** |
|  | Domestic_chicken | Domestic_turkey | 17.64 | 0.77 |
|  | Wild_Ans | OOS | 8.54 | 0.62 |
|  | **Domestic_turkey** | **Wild_non-Ans** | **7.83** | **0.60** |

**^a^ OOS: Outside of Spain.**
