# Supplementary material for: Wild birds drive the introduction, maintenance, and spread of H5N1 clade 2.3.4.4b high pathogenicity avian influenza viruses in Spain, 2021–2022
Source: Virus Evol. 2026 Jan 30;12(1):veag006. doi: 10.1093/ve/veag006 (PMC12931561; doi:10.1093/ve/veag006)
Supplement: supplementary-material_veag006 [file supplementary-material_veag006.zip › Supplementary_Table_S7_new_KBD_veag006.docx]

**Supplementary Table S7. Transmission matrix of Bayesian stochastic search variable selection analysis of geographic location trait.** Overlapping transitions are shown in bold

| Dataset | From | To | Bayes factor | Posterior Probability |
| --- | --- | --- | --- | --- |
| Complete dataset (n=231) | **OOS**^a^ | **NORTH-WEST** | **6325.52** | **1.00** |
|  | **OOS** | **NORTH** | **1486.27** | **1.00** |
|  | **OOS** | **NORTH-EAST** | **1125.22** | **1.00** |
|  | OOS | SOUTH | 1065.72 | 1.00 |
|  | **SOUTH-WEST** | **OOS** | **461.75** | **0.99** |
|  | **SOUTH-WEST** | **SOUTH** | **361.37** | **0.99** |
|  | **NORTH-WEST** | **SOUTH** | **94.83** | **0.95** |
|  | OOS | CENTRAL | 65.08 | 0.93 |
|  | **CENTRAL** | **NORTH-EAST** | **52.16** | **0.91** |
|  | **CENTRAL** | **NORTH-WEST** | **36.03** | **0.87** |
|  | **NORTH** | **NORTH-WEST** | **21.12** | **0.80** |
|  | OOS | SOUTH-WEST | 15.76 | 0.75 |
|  | **SOUTH-WEST** | **CENTRAL** | **7.26** | **0.58** |
| Down-sampled dataset (n=119) | **SOUTH-WEST** | **SOUTH** | **213.33** | **0.98** |
|  | **OOS** | **NORTH** | **124.17** | **0.96** |
|  | **OOS** | **NORTH-EAST** | **104.03** | **0.95** |
|  | **NORTH** | **NORTH-WEST** | **30.14** | **0.85** |
|  | **CENTRAL** | **NORTH-WEST** | **26.69** | **0.83** |
|  | **NORTH-WEST** | **SOUTH** | **26.29** | **0.83** |
|  | **CENTRAL** | **NORTH-EAST** | **25.16** | **0.83** |
|  | **OOS** | **NORTH-WEST** | **12.15** | **0.70** |
|  | **SOUTH-WEST** | **CENTRAL** | **7.10** | **0.57** |
|  | **SOUTH-WEST** | **OOS** | **6.28** | **0.54** |

^a^ OOS: Outside of Spain.
