# Supplementary figures and images for: Wild birds drive the introduction, maintenance, and spread of H5N1 clade 2.3.4.4b high pathogenicity avian influenza viruses in Spain, 2021–2022
Source: Virus Evol. 2026 Jan 30;12(1):veag006. doi: 10.1093/ve/veag006 (PMC12931561; doi:10.1093/ve/veag006)

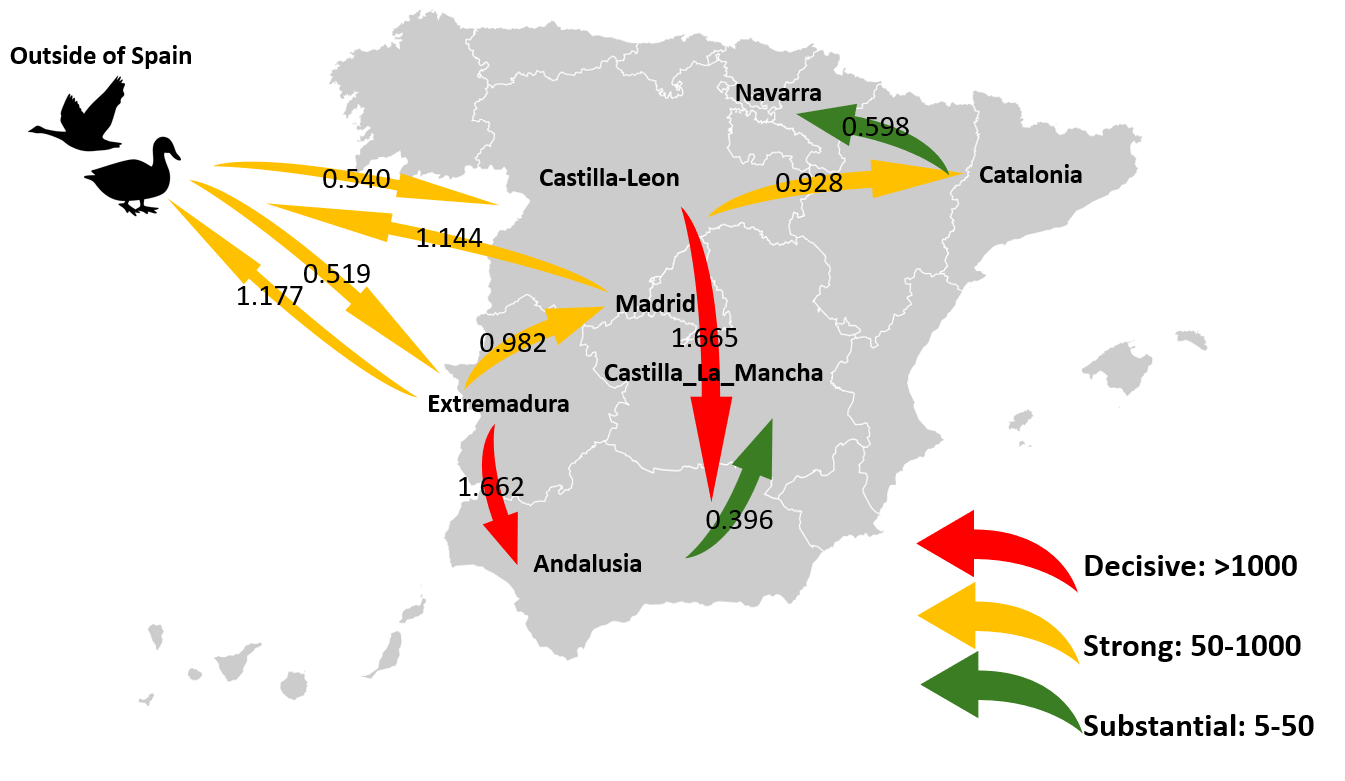

Supplement: supplementary-material_veag006 [file supplementary-material_veag006.zip › SupFigS4_fix_veag006.PNG]

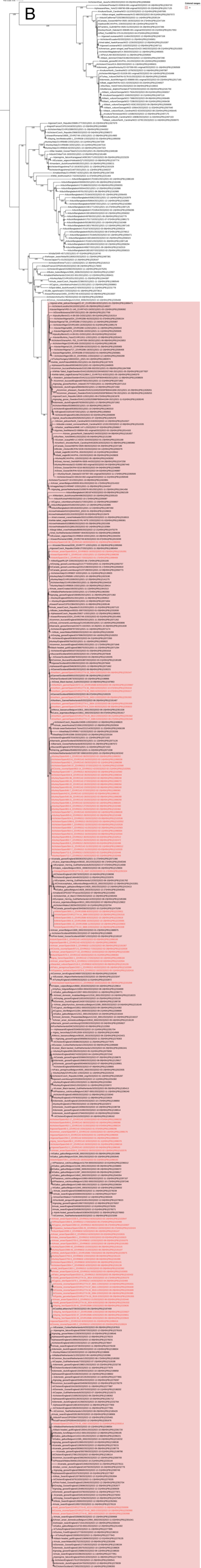

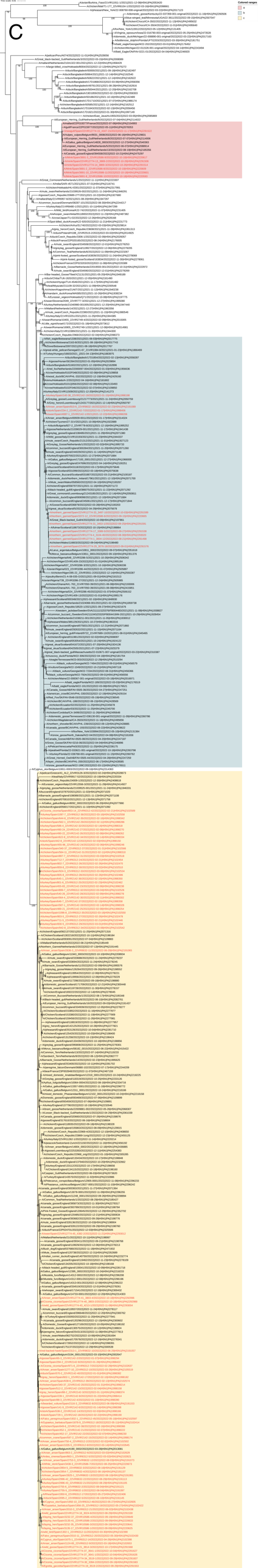

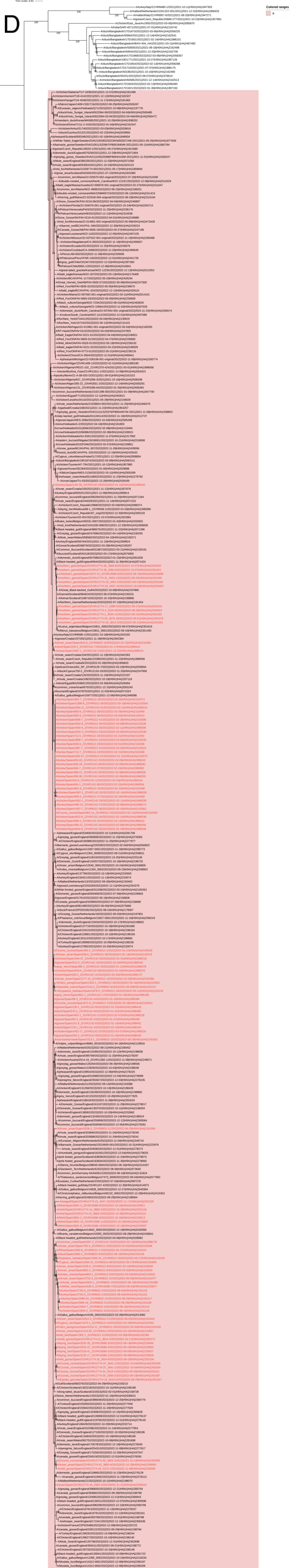

E

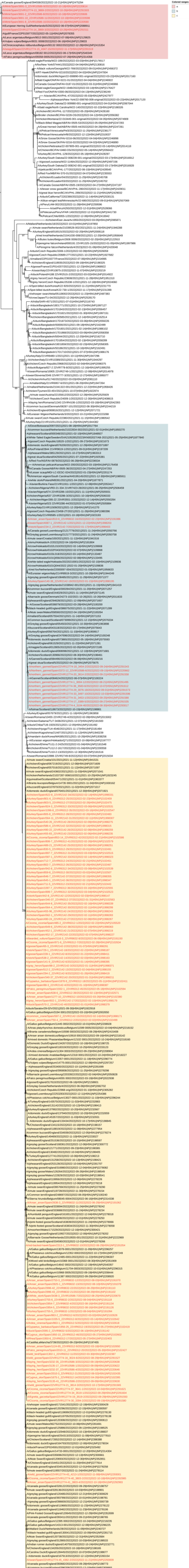

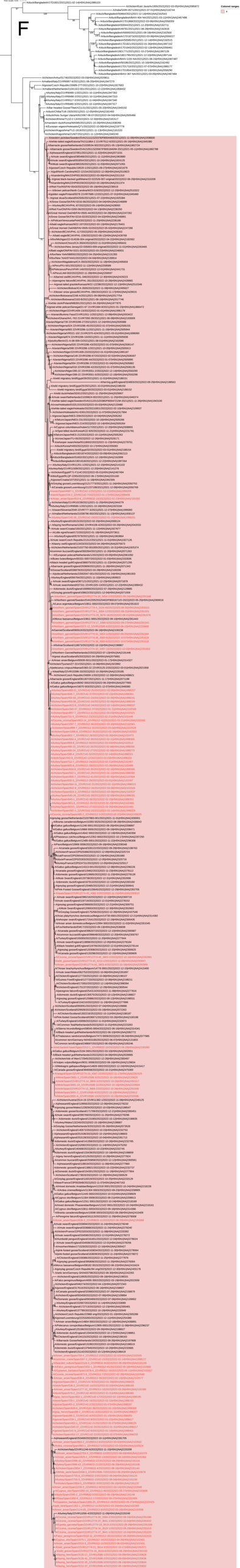

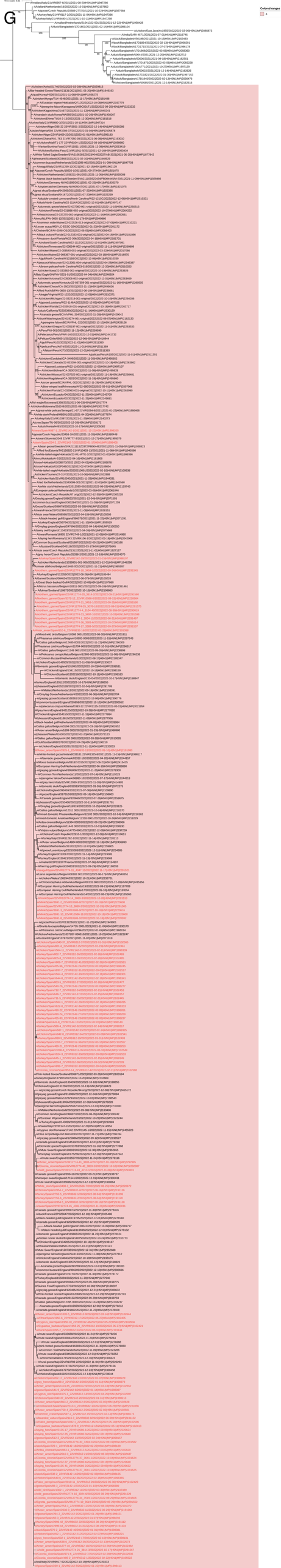

H

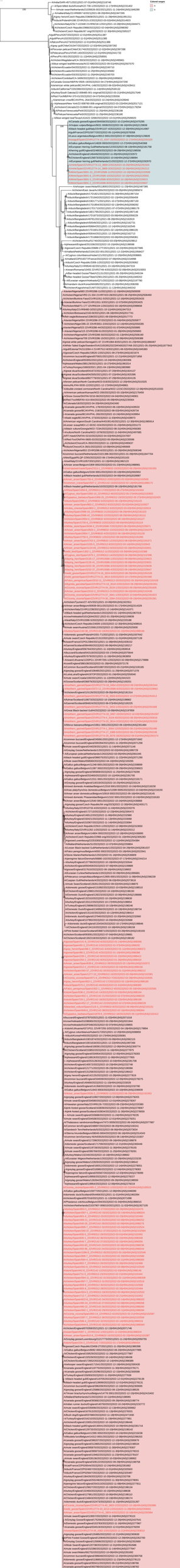

Supplement: supplementary-material_veag006 [file supplementary-material_veag006.zip › Supplementary_Figure_S1_veag006.pdf]

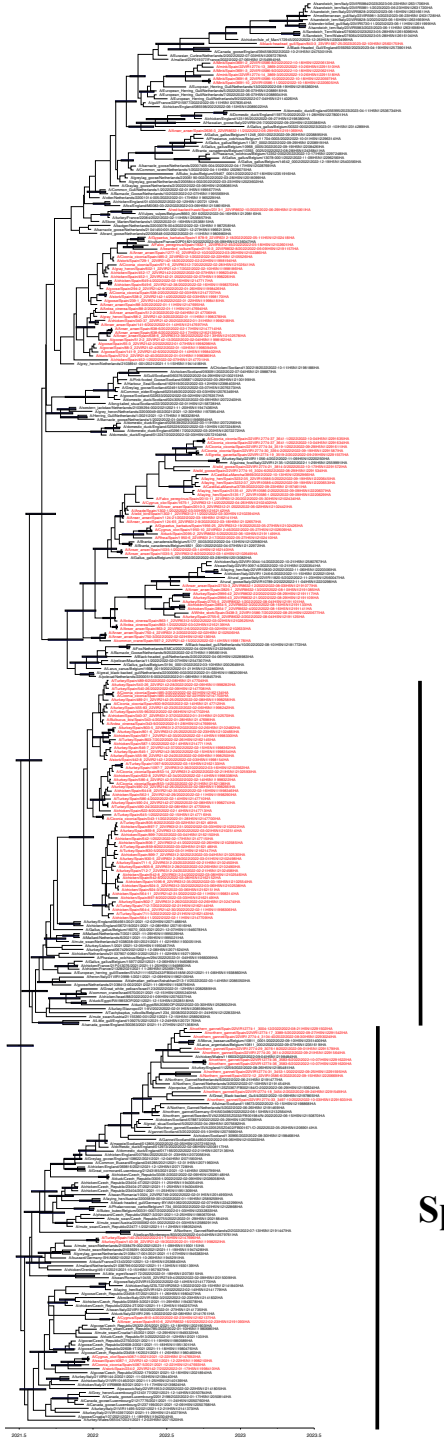

**Spain-  
2.2.1**

**Spain-  
2.2**

**Spain-2**

**Spain-2.1**

**Spain-1**

Supplement: supplementary-material_veag006 [file supplementary-material_veag006.zip › Supplementary_Figure_S2_veag006.pdf]

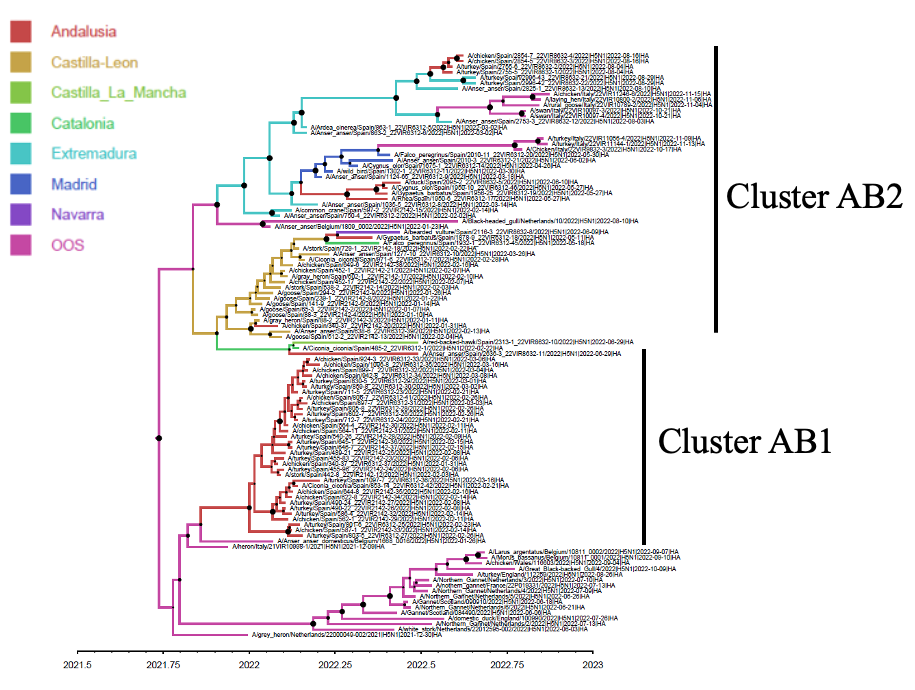

Supplement: supplementary-material_veag006 [file supplementary-material_veag006.zip › Supplementary_Figure_S3A_veag006.png]

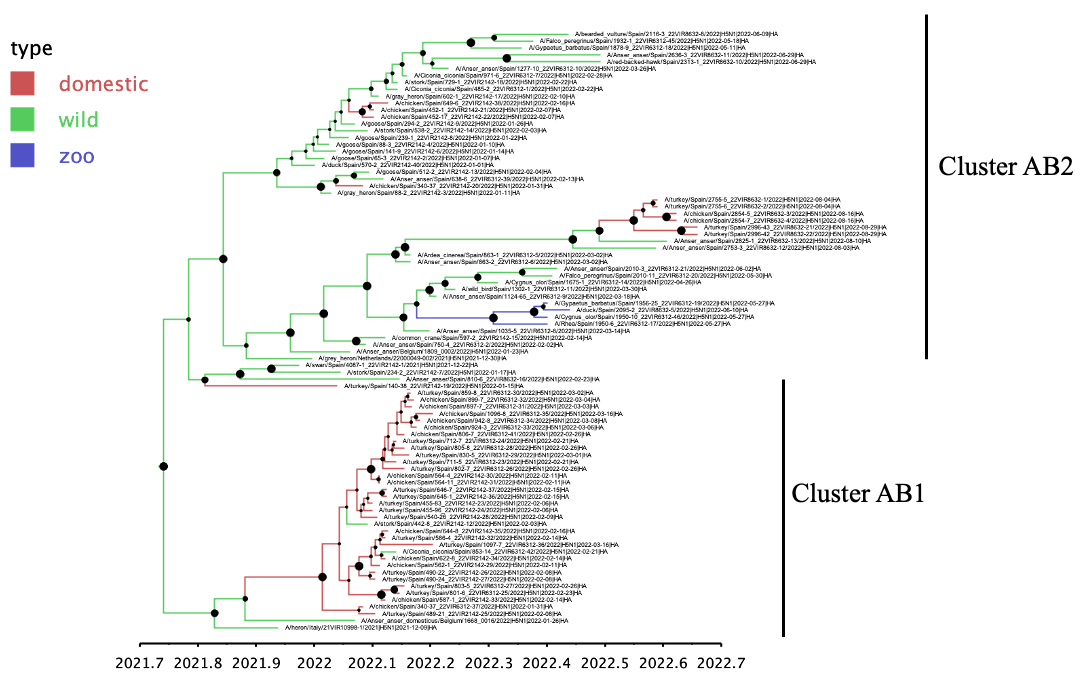

Supplement: supplementary-material_veag006 [file supplementary-material_veag006.zip › Supplementary_Figure_S3B_veag006.png]

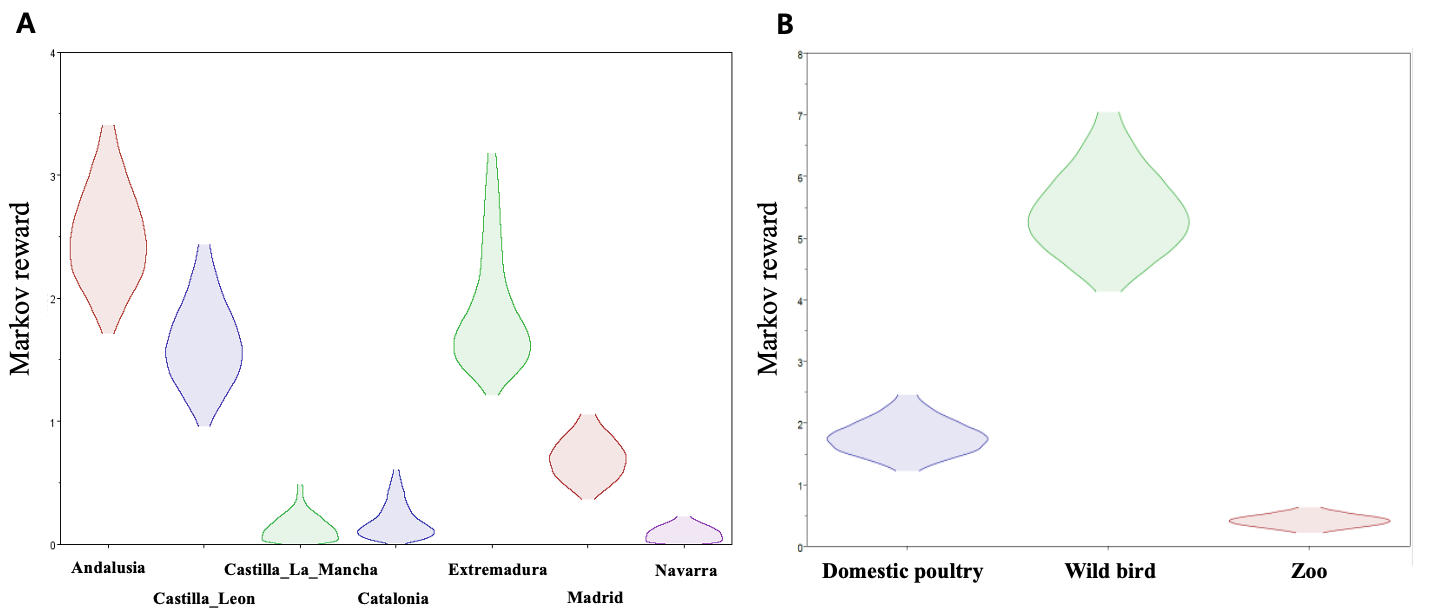

Supplement: supplementary-material_veag006 [file supplementary-material_veag006.zip › Supplementary_Figure_S5_veag006.png]

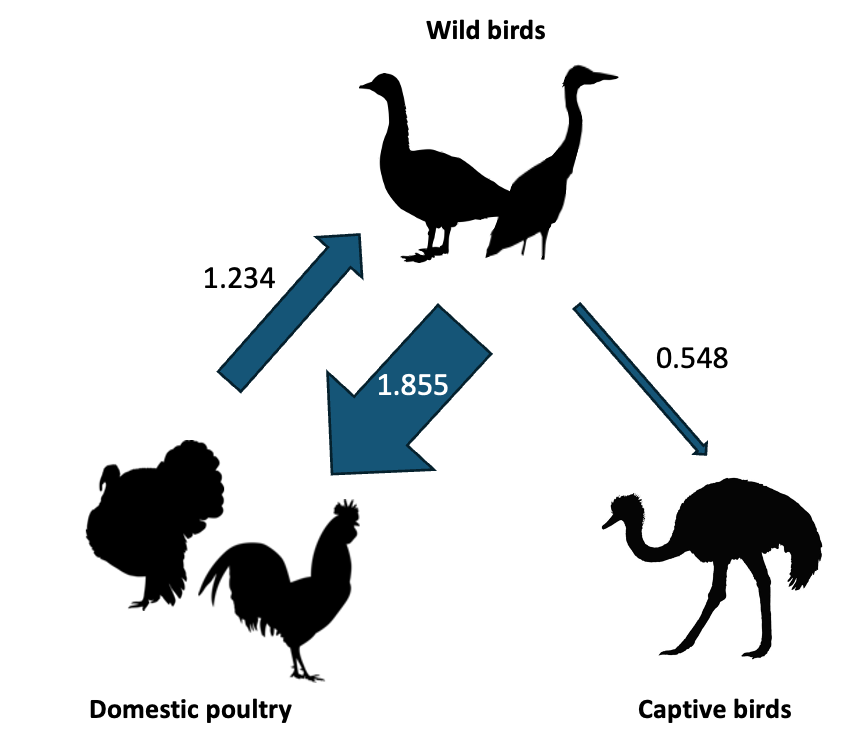

Supplement: supplementary-material_veag006 [file supplementary-material_veag006.zip › Supplementary_Figure_S6_veag006.png]

A

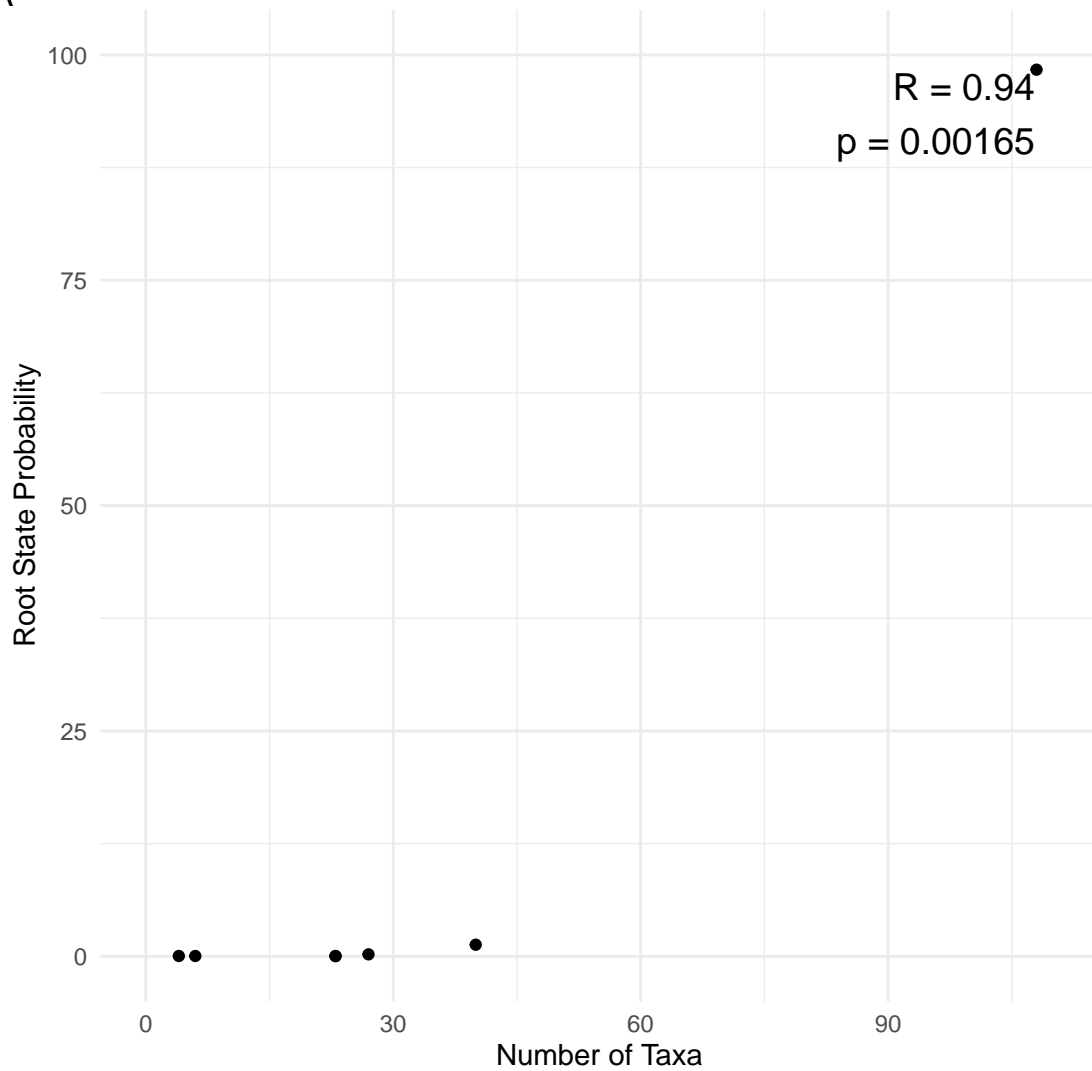

B

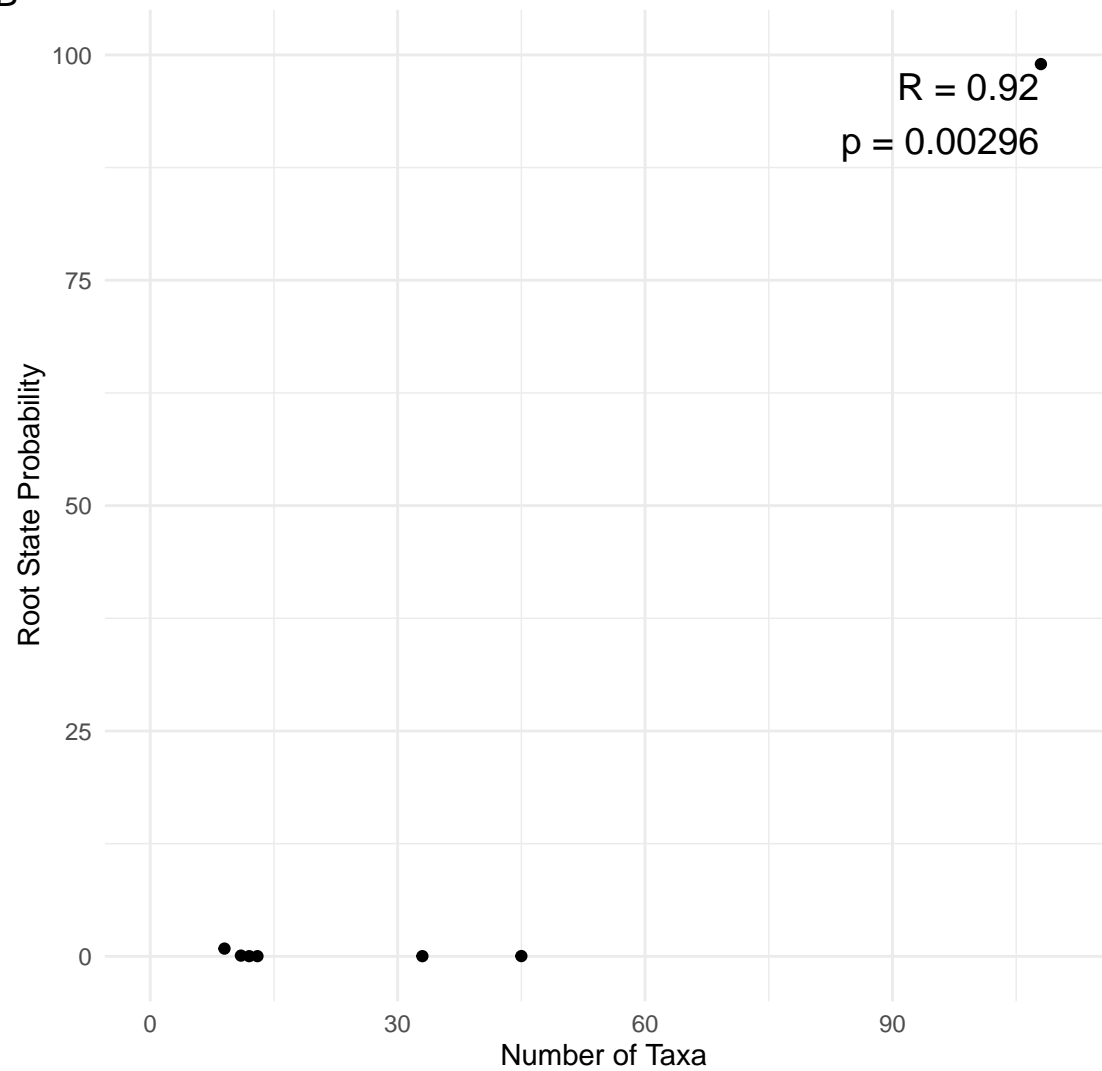

Supplement: supplementary-material_veag006 [file supplementary-material_veag006.zip › Supplementary_Figure_S7_new_veag006.pdf]

A

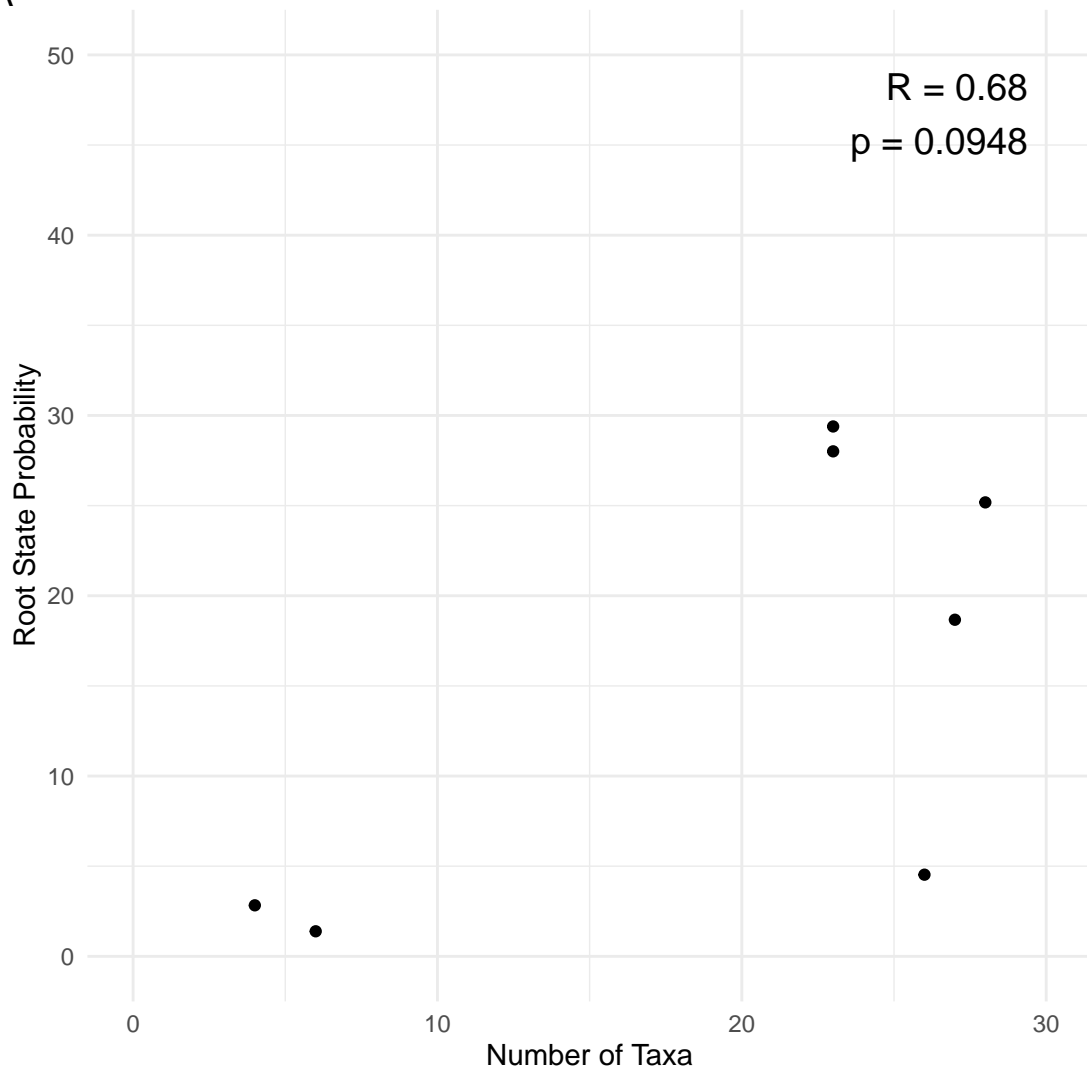

B

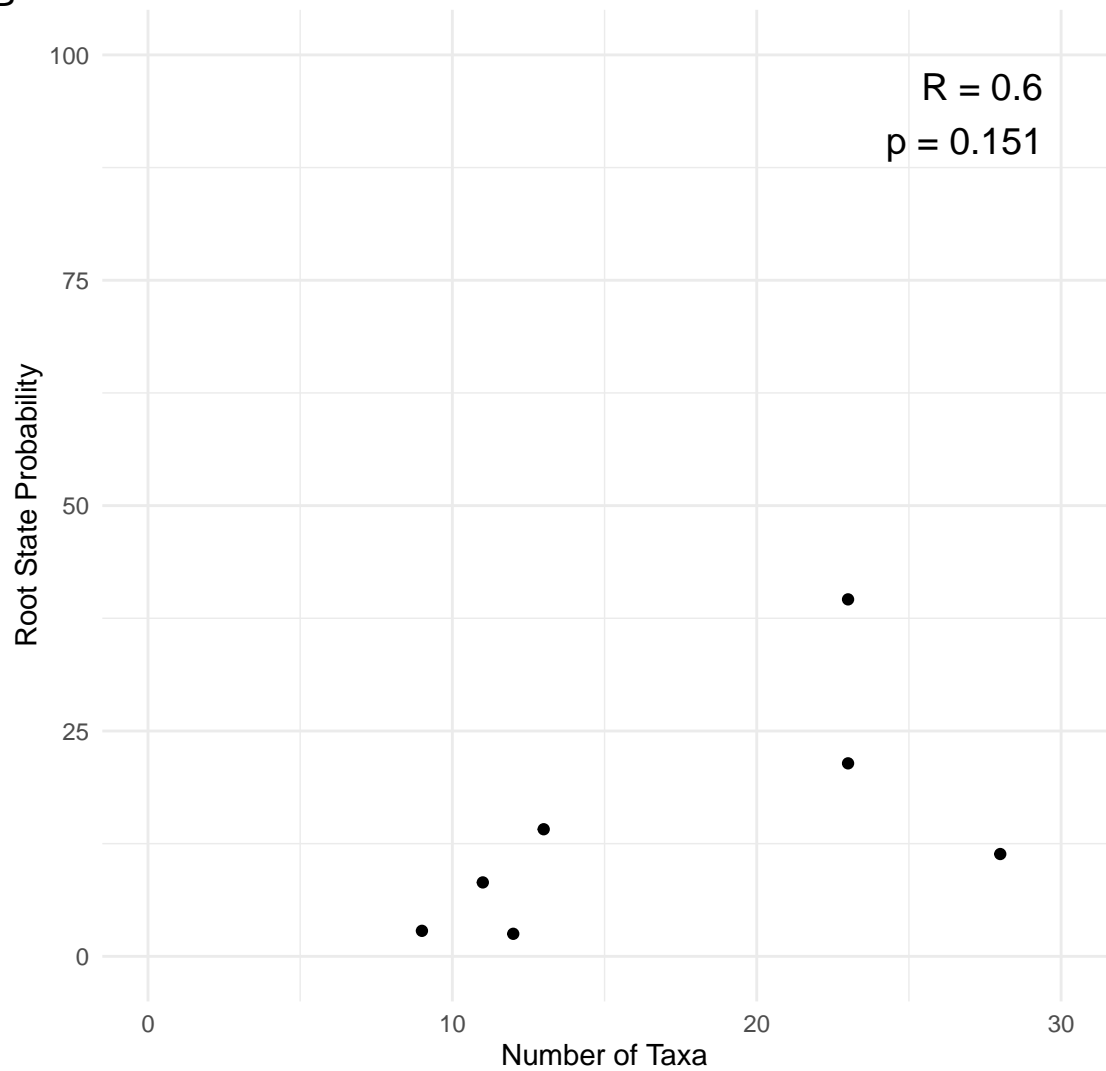

Supplement: supplementary-material_veag006 [file supplementary-material_veag006.zip › Supplementary_Figure_S8_new_veag006.pdf]
